# Porcine reproductive and respiratory syndrome virus exploits ESCRT-II subunit EAP20 for entry and replication

**DOI:** 10.1128/jvi.02109-25

**Published:** 2026-03-10

**Authors:** Longxiang Zhang, Yan Jiang, Rui Li, Mengjie Wang, Xinrong Wang, Junhai Zhu, Nan Yan, Songlin Qiao, Rui Li, Yue Wang

**Affiliations:** 1 College of Veterinary Medicine, Southwest University 26463, Chongqing, China; 2 Institute for Animal Health, Henan Academy of Agricultural Sciences 74728, Zhengzhou, Henan, China; 3 National Center of Technology Innovation for Pigs, Chongqing, China; University of Michigan Medical School, Ann Arbor, Michigan, USA

**Keywords:** PRRSV, ESCRT, EAP20, entry, replication, double-membrane vesicles

## Abstract

Porcine reproductive and respiratory syndrome (PRRS), caused by PRRS virus (PRRSV), is a major viral disease that poses a serious threat to the global swine industry. Although progress has been made in understanding its life cycle, the molecular mechanisms underlying PRRSV entry and replication remain incompletely understood. Multiple RNA viruses hijack the endocytic sorting complex required for transport (ESCRT) machinery to orchestrate various stages during infection. In the current study, we identified ESCRT-II subunit ELL-associated protein 20 (EAP20) as an important host factor involved in PRRSV entry and replication. Mechanistically, EAP20 participated in the transport of internalized PRRSV particles to early endosomes via the clathrin-mediated endocytosis pathway. During replication, EAP20 interacted with PRRSV nonstructural protein (Nsp) 2, Nsp5, and Nsp9. Specifically, EAP20 anchored the core replicase Nsp9 on the perinuclear endoplasmic reticulum (ER) and coordinated with the transmembrane proteins Nsp2/Nsp5 to form ER-derived double-membrane vesicles. Collectively, our findings demonstrate that PRRSV exploits EAP20 for viral entry and replication, highlighting EAP20 as a novel proviral factor and a potential antiviral target.

**IMPORTANCE:**

PRRSV remains one of the most economically significant pathogens in the global swine industry. Current control strategies are largely hindered because PRRSV pathogenesis has not been fully elucidated. In this study, we identified EAP20, a core subunit of ESCRT-II, as a multifaceted proviral factor that participated in PRRSV entry and replication. These findings provide new insights into the interplay between PRRSV and the host ESCRT machinery, laying a foundation for the development of more effective strategies for PRRS control.

## INTRODUCTION

Porcine reproductive and respiratory syndrome (PRRS), also termed “blue-ear disease,” is a highly contagious viral disease (1). Its main clinical manifestations encompass reproductive failure in sows of the late-term gestation, characterized by abortion, premature delivery, stillbirth, and a mummified fetus, as well as acute respiratory distress in pigs across all ages (2). Since its emergence in the 1980s, PRRS has become widespread across North America, Europe, and Asia, causing substantial economic losses to the global swine production (3, 4). In the United States, annual economic losses attributed to PRRS are estimated to be up to $1.2 billion (5).

PRRS virus (PRRSV), the causative agent of PRRS, is an enveloped, single-stranded, positive-sense RNA virus classified within the order *Nidovirales*, the family *Arteriviridae*, and the genus *Betaarterivirus* (6). The PRRSV genome encodes at least 16 nonstructural proteins (Nsps) and eight structural proteins (7, 8). PRRSV exhibits a highly restricted host cell tropism *in vitro*, primarily targeting porcine alveolar macrophages (PAMs), African green monkey kidney epithelial cell line MA-104, and its clonal derivative MARC-145 (9, 10). As an obligate intracellular pathogen, PRRSV usurps various host cellular components, such as endosome trafficking and membrane-remodeling machinery, to complete its life cycle, including attachment, entry, replication, assembly, and release (11). Despite significant advancements in understanding the PRRSV life cycle, the molecular mechanisms are not fully characterized (12–15). This gap in knowledge hampers the development of effective control strategies for PRRS.

The endocytic sorting complex required for transport (ESCRT) system is an evolutionarily conserved molecular machinery found in all eukaryotic cells. It comprises five core subcomplexes, ESCRT-0, -I, -II, -III, and vacuolar protein sorting 4 (VPS4), along with several associated auxiliary proteins (16). These multisubunit complexes play roles in sorting membrane proteins and manipulating lipid bilayer scission (17, 18). ELL-associated protein 20 (EAP20), also known as vacuolar protein sorting 25 (VPS25), is a core component of ESCRT-II and forms a heterotetrameric complex with EAP30 and EAP45 (19). EAP20 has been identified as a proviral factor for a variety of viruses, such as rotavirus, hepatitis B virus, human immunodeficiency virus, and enterovirus 71 (20–24). However, the specific function of EAP20 in the PRRSV life cycle remains unexplored.

In this study, we initially demonstrated that EAP20 plays an important role in PRRSV proliferation through multiple experimental approaches. We subsequently found that EAP20 was involved in PRRSV internalization by trafficking viral particles to early endosomes (EEs) via the clathrin-mediated endocytosis (CME) pathway. Furthermore, we revealed that EAP20 interacted with PRRSV Nsps to localize the viral replicase on the perinuclear endoplasmic reticulum (ER) and form ER-derived double-membrane vesicles (DMVs).

## RESULTS

### Identification of ESCRT-II subunits required for PRRSV proliferation

**Fig 1:**
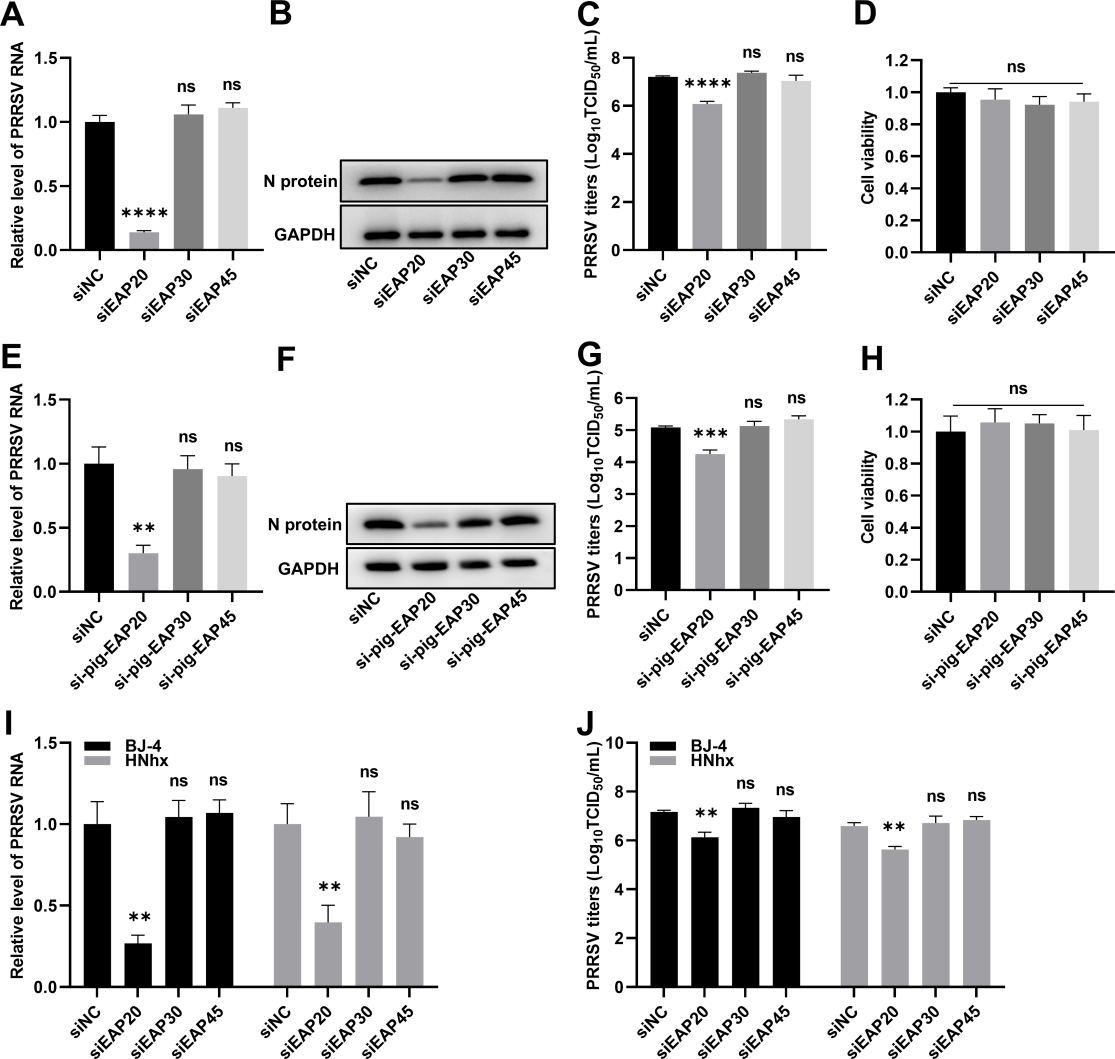
Identification of ESCRT-II subunits required for PRRSV proliferation. (**A–D**) Silencing the ESCRT-II subunit EAP20 impeded PRRSV proliferation in MARC-145 cells. The MARC-145 cells seeded in 24-well plates were transfected with siRNA duplexes targeting ESCRT-II subunits (*EAP20/EAP30/EAP45*) or the scrambled siRNA (siNC) for 36 h, and then infected with PRRSV at an MOI of 0.5. At 24 hpi, the infected cells were harvested for determining the total PRRSV RNA abundance using RT-qPCR with the specific primers targeting the open reading frame 7 (ORF7) of the viral genome (**A**), N protein expression levels using IB with rabbit anti-PRRSV N pAbs (**B**), and extracellular progeny virus titers by assessing TCID_50_ (**C**). (**D**) The viability of MARC-145 cells was assessed using the CCK-8 assay upon transfection with the specified ESCRT-II subunit siRNAs for 60 h. The cells transfected with siNC served as the control group, with their viabilities normalized to 1.0 for comparative analysis. (**E–H**) Silencing the ESCRT-II subunit EAP20 impeded PRRSV proliferation in CRL-2843-CD163 cells. The CRL-2843-CD163 cells were treated similarly to MARC-145 cells as described above. After the samples were collected, the PRRSV RNA abundance (**E**), N protein expression levels (**F**), extracellular progeny virus titers (**G**), and cell viabilities (**H**) were determined. (**I, J**) Silencing the ESCRT-II subunit EAP20 attenuated the infectivity of different PRRSV strains. The MARC-145 cells transfected with siEAP20, siEAP30, or siEAP45 were infected with 0.5 MOI of PRRSV strain BJ-4 or HNhx. At 24 hpi, the samples were harvested to quantify the PRRSV RNA abundance (**I**) and extracellular progeny virus titers (**J**) by the aforementioned methods. Data are shown as means ± SEM from three independent experiments. **, *P* < 0.01; ***, *P* < 0.001; ****, *P* < 0.0001. ns, not significant, *P* > 0.05. *P*-values were analyzed by one-way ANOVA.

Our previous small-interference RNA (siRNA)-screening experiments revealed that certain subcomplexes of the ESCRT system were required for PRRSV proliferation (25). To specifically determine the contribution of the ECSRT-II complex, the specific siRNA duplexes targeting EAP20, EAP30, or EAP45 were separately transfected into MARC-145 cells. Following 36 h of transfection, the cells were infected with PRRSV strain HN07-1 for 24 h, and both the cell lysates and the supernatants were harvested to assess viral infectivity by monitoring the intracellular viral RNA abundance, nucleocapsid (N) protein expression levels, and extracellular progeny virus titers. Quantitative real-time PCR (RT-qPCR), immunoblotting (IB), and 50% tissue culture infected dose (TCID_50_) assays consistently indicated that depletion of EAP20 rather than EAP30 or EAP45 significantly hindered PRRSV proliferation (Fig. 1A through C). Cell viability assays confirmed that transfection with these siRNAs was non-cytotoxic to MARC-145 cells (Fig. 1D). To verify these findings, we repeated the experiments in CRL-2843-CD163 cells, an immortalized PAM cell line stably expressing the PRRSV receptor CD163 (26). Similarly, *EAP20* knockdown resulted in a reduction of PRRSV RNA levels, N protein expression, and viral titers (Fig. 1E through H). To assess whether this requirement for EAP20 was strain-independent, MARC-145 cells were transfected with siRNAs targeting ESCRT-II subunits and subsequently infected with two additional PRRSV strains, BJ-4 and HNhx. In both cases, *EAP20* knockdown led to a decrease in viral RNA loads and progeny virus titers, whereas depletion of EAP30 or EAP45 had minimal effect (Fig. 1I and J). Taken together, these results identify ESCRT-II subunit EAP20 required for PRRSV proliferation across different viral strains and host cells.

### PRRSV exploits EAP20 to enhance viral proliferation

**Fig 2:**
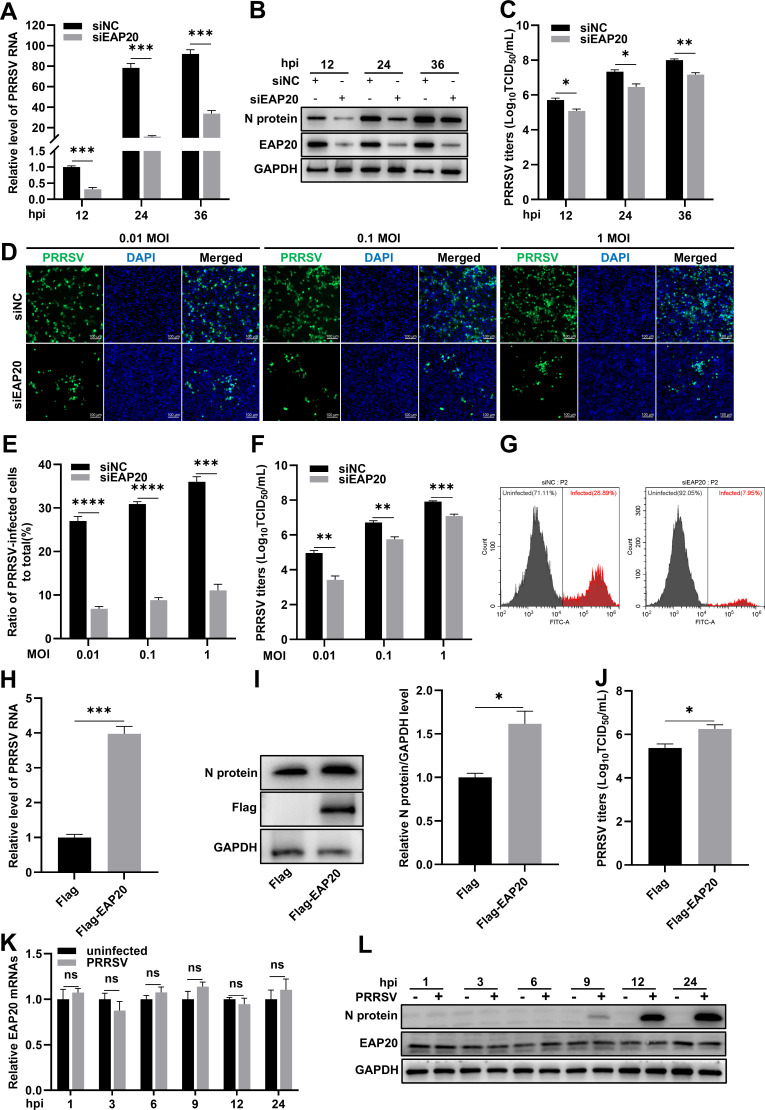
PRRSV exploits EAP20 to enhance viral proliferation. (**A–C**) Knockdown of EAP20 suppressed PRRSV proliferation. The MARC-145 cells transfected with siNC or siEAP20 were infected with PRRSV (MOI = 0.1) at 36 h post-transfection (hpt). The infected cells were harvested at the indicated time points (12, 24, or 36 hpi) for subsequent analyses. (**A**) The relative PRRSV RNA abundance was detected by RT-qPCR. (**B**) The PRRSV N protein and endogenous EAP20 levels were evaluated by IB. (**C**) The PRRSV titers in supernatants were monitored by virus titration assay. (**D–F**) Knockdown of EAP20 inhibited PRRSV proliferation at different MOIs. The MARC-145 cells transfected with siNC or siEAP20 for 36 h were infected with PRRSV at different MOIs (0.01, 0.1, or 1), and the indicated samples were collected at 24 hpi for the following experiments. (**D**) The intracellular expression level of viral N protein was observed by IFA with a mouse anti-PRRSV N MAb. Nuclei were dyed blue with DAPI, and the scale bars were equal to 100 μm. (**E**) The proportion of infected cells (with green fluorescence) to the total cells (with blue fluorescence) was calculated using ImageJ software to represent the infectivity of PRRSV. (**F**) The progeny virus titers in supernatants were quantified by assessing TCID_50_. (**G**) MARC-145 cells were transfected in a similar manner as described above and then infected with PRRSV at 0.1 MOI. At 24 hpi, the infected cells were digested, fixed, and stained for detection of PRRSV infectivity by FCM. (**H–J**) Overexpression of EAP20 promoted PRRSV proliferation. MARC-145 cells were transfected with Flag-EAP20 or empty vectors (Flag) for 24 h before inoculation with PRRSV at an MOI of 0.1 for 24 h. (**H**) The cells were harvested and lysed for the quantification of PRRSV RNA abundance by RT-qPCR. (**I**) The cells were collected to evaluate the expression levels of viral N protein and EAP20 by IB, and the protein band intensities were quantified using ImageJ software. The ratio of N protein/GAPDH in the vector-transfected group was normalized to 1.0. (**J**) The extracellular supernatants were harvested for the determination of the viral titers by titration assay. (**K, L**) EAP20 expression was stable during PRRSV infection. The MARC-145 cells were infected or uninfected with PRRSV (MOI = 1) and collected at the indicated time points (1, 3, 6, 9, 12, or 24 hpi) for the determination of EAP20 mRNA abundance by RT-qPCR (**K**) and its protein levels by IB (**L**). Data were shown as means ± SEM from three independent experiments. *, *P* < 0.05; **, *P* < 0.01; ***, *P* < 0.001; ****, *P* < 0.0001. ns, not significant, *P* > 0.05. *P*-values were analyzed by unpaired two-tailed Student’s *t*-tests.

To further elucidate the role of EAP20 in PRRSV proliferation, we knocked down EAP20 in MARC-145 cells and examined its impact on viral replication kinetics. RT-qPCR results showed that *EAP20* knockdown significantly decreased PRRSV RNA abundance by 68.9%, 85.5%, and 63.3% at 12, 24, and 36 h post-infection (hpi), respectively (Fig. 2A). Consistently, IB analysis revealed that the PRRSV N protein expression was decreased by 63.5%, 75.8%, and 52.4% at the corresponding time points (Fig. 2B). Moreover, TCID_50_ data revealed that viral titers were dropped by 5.1-fold, 7.6-fold, and 6.9-fold following *EAP20* depletion (Fig. 2C). Indirect immunofluorescence assay (IFA) analysis further confirmed that PRRSV infectivity was decreased by 74.6%, 71.4%, and 69.2% in the *EAP20* knockdown cells at different multiplicities of infection (MOIs; Fig. 2D and E). In accordance with this observation, the viral titration assay exhibited significantly lower viral titers in the *EAP20* knockdown cells, which were 31.6-fold, 8.9-fold, and 6.6-fold lower than those in the control cells (Fig. 2F). Additionally, flow cytometry (FCM) results revealed a marked decline in the proportion of PRRSV-positive cells, decreasing from 28.89% to 7.95% following *EAP20* silencing (Fig. 2G). Conversely, overexpression of EAP20 enhanced PRRSV proliferation, as evidenced by increased viral RNA abundance (3.9-fold, Fig. 2H), elevated N protein expression (61.6%, Fig. 2I), and higher progeny virus titers (7.4-fold, Fig. 2J).

To comprehend whether EAP20 was modulated during PRRSV proliferation, we analyzed its transcriptional and protein levels at different time points post-infection using RT-qPCR and IB. As shown in Fig. 2K and L, neither the mRNA nor the protein expression of EAP20 exhibited significant changes throughout the course of infection, indicating that PRRSV proliferation does not alter EAP20 expression levels. Together, these results demonstrate that PRRSV utilizes EAP20 to facilitate its proliferation.

### EAP20 facilitates PRRSV entry and replication

**Fig 3:**
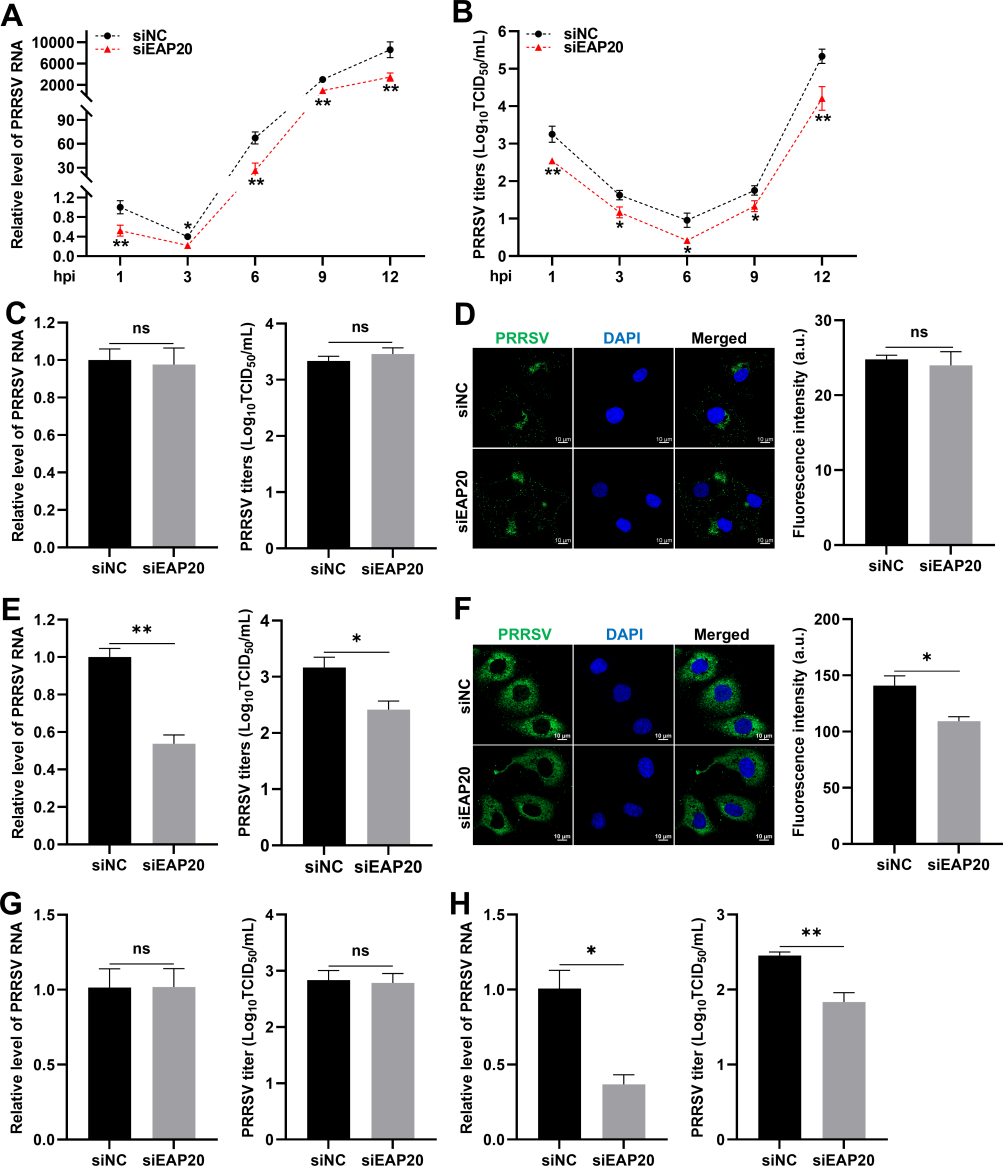
EAP20 is involved in PRRSV internalization. (**A and B**) Knockdown of EAP20 affected PRRSV proliferation in the early stage. The MARC-145 cells transfected with siNC or siEAP20 were infected with 1 MOI PRRSV, and the infected cells were collected at the indicated time points (1, 3, 6, 9, or 12 hpi) for subsequent studies. (**A**) The relative intracellular PRRSV RNA abundance at the indicated time points was detected by RT-qPCR. (**B**) The cells and supernatants were collected together and repeatedly freeze-thawed at −80°C three times. After centrifugation, the resulting samples were used to evaluate PRRSV titers by titration assay. (**C and D**) Silencing EAP20 did not affect PRRSV attachment. The MARC-145 cells transfected with siNC or siEAP20 were infected with PRRSV at 10 MOI and incubated at 4°C for 1 h. After binding, the infected cells were harvested and washed five times with pre-chilled PBS to completely remove the unadsorbed viruses on the cell surface. (**C**) The RNA levels and titers of the cell-bound PRRSV were determined using RT-qPCR and titration assay, respectively. (**D**) Cell-bound PRRSV particles were visualized by confocal microscopy with mouse anti-GP5 MAb and Alexa Fluor 488-donkey anti-mouse IgG pAbs. The intensity of total green fluorescence was calculated using ImageJ software. These images represent at least 10 analyzed cells from three independent experiments. Nuclei were stained blue with DAPI, and scale bars were indicated as 10 μm. (**E and F**) Silencing EAP20 hindered PRRSV internalization. The MARC-145 cells transfected with siNC or siEAP20 were first subjected to the same treatments as for viral attachment and then transferred to 37°C for another 1 h. After discarding the medium, the cells were washed thoroughly with pre-chilled PBS containing proteinase K to remove the adsorbed viruses. After the washing process, the RNA levels and titers of the entered PRRSV (**E**) and the fluorescence signals of N protein (**F**) were measured in a similar manner as described above, respectively. (**G and H**) Silencing EAP20 did not affect PRRSV attachment but inhibited its internalization in CRL-2843-CD163 cells. The CRL-2843-CD163 cells transfected with siNC or si-pig-EAP20 were infected with PRRSV at an MOI of 10. PRRSV entry and internalization experiments were performed as described for C and E. Data are shown as means ± SEM from three independent experiments. *, *P* < 0.05; **, *P* < 0.01. ns, not significant, *P* > 0.05. *P*-values were analyzed by unpaired two-tailed Student’s *t*-tests.

To determine how it participated in the viral proliferation, we investigated the effect of *EAP20* knockdown on the specific stages of PRRSV life cycle. Time-course analysis revealed that knockdown of EAP20 significantly reduced intracellular PRRSV RNA abundance and progeny virion titers as early as 1 hpi (Fig. 3A and B), suggesting that EAP20 acts during an early stage of infection.

The processes of attachment and subsequent entry represent critical early events for the virus to establish efficient infection. Therefore, we first explored whether EAP20 influenced viral attachment. The MARC-145 cells transfected with siNC or EAP20-targeted siRNA (siEAP20) were infected with PRRSV (MOI = 10) at 4°C for 1 h to allow viral binding without entry, followed by the removal of unbound virions. Viral attachment was assessed by RT-qPCR, virus titration, and confocal microscopy. No significant differences were observed between the siNC- and siEAP20-transfected cells in PRRSV RNA abundance, viral titers, or total fluorescence intensity of glycoprotein 5 (GP5, Fig. 3C and D), indicating that EAP20 does not affect viral attachment. Subsequently, we detected whether EAP20 was involved in PRRSV entry. After viral binding at 4°C, the cells were transferred to 37°C for 1 h to allow entry. RT-qPCR, virus titration, and confocal microscopy consistently showed that knockdown of EAP20 diminished the internalization of PRRSV particles (Fig. 3E and F), suggesting that EAP20 is required for the viral entry. Furthermore, we confirmed in the CRL-2843-CD163 cells that knocking down EAP20 also effectively inhibited PRRSV entry without affecting its attachment (Fig. 3G and H).

**Fig 4:**
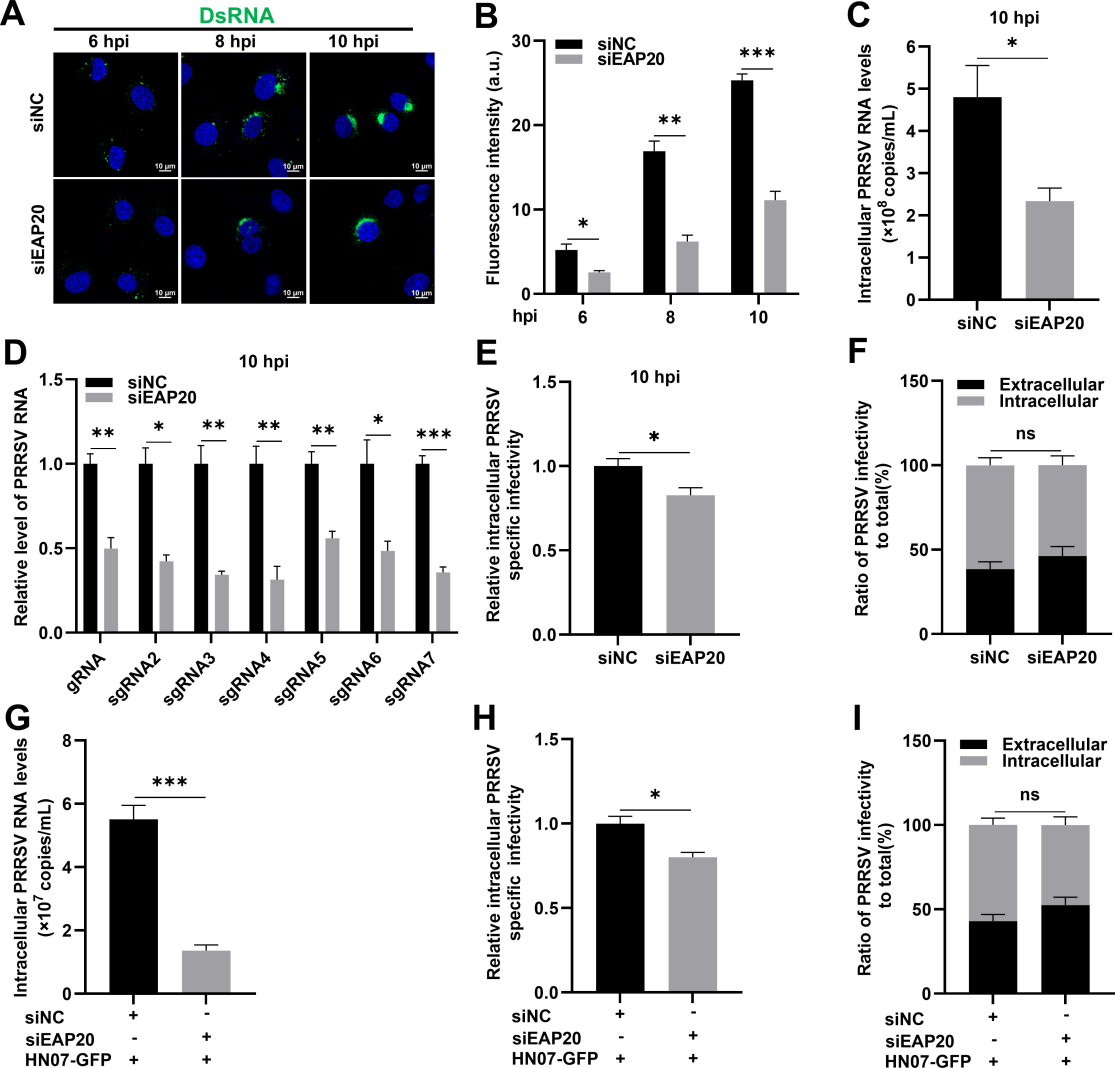
EAP20 is involved in PRRSV replication. (**A**) The MARC-145 cells transfected with siNC or siEAP20 were infected with PRRSV (MOI = 1) and incubated at 37°C for 6, 8, or 10 h. The cells were fixed and subjected to incubation with mouse anti-dsRNA MAb, followed by fluorescent secondary antibodies. Confocal microscopy was used to visualize the abundance of viral dsRNA. Nuclei were stained blue with DAPI, and the scale bars were indicated as 10 μm. These images represent at least 10 analyzed cells from three independent experiments. (**B**) The total fluorescence intensity of dsRNA was calculated using ImageJ software. (**C–F**) Knockdown of EAP20 affected PRRSV replication in MARC-145 cells. The MARC-145 cells transfected with siNC or siEAP20 were infected with PRRSV (MOI = 1) at 37°C for 10 h. (**C**) The intracellular PRRSV RNA copies were quantified by RT-qPCR within the first life cycle. (**D**) The abundance of PRRSV gRNA and all sgRNAs was evaluated by RT-qPCR. (**E**) PRRSV assembly efficiency was indicated by the relative intracellular specific infectivity and determined by comparing the intracellular viral infectivity (TCID_50_/mL) with the intracellular PRRSV RNA levels. (**F**) PRRSV release efficiency was calculated as the ratio of intracellular and extracellular infectivity relative to the total infectivity. (**G–I**) Knockdown of EAP20 affected PRRSV replication in HEK-293T cells. The HEK-293T cells with or without *EAP20* knockdown were transfected with the infectious clone of PRRSV strain HN07-1 (HN07-GFP) for 36 h. The cells were collected and assessed for PRRSV RNA copies (**G**), assembly efficiency (**H**), and release efficiency (**I**) using the same methods as previously mentioned in MARC-145 cells. Data are shown as means ± SEM from three independent experiments. *, *P* < 0.05; **, *P* < 0.01; ***, *P* < 0.001; ns, not significant, *P* > 0.05. *P*-values were analyzed by unpaired two-tailed Student’s *t*-tests.

Upon entering the cell, PRRSV typically releases its genomic RNA into the cytoplasm to initiate replication. Thus, we subsequently investigated whether EAP20 was involved in PRRSV post-entry stages. To assess the role of EAP20 in PRRSV replication, the infected cells were analyzed at 6, 8, and 10 hpi. Immunofluorescence staining for double-stranded RNA (dsRNA, the intermediate products of RNA synthesis, served as a hallmark of positive-strand RNA virus replication [27]) showed that knockdown of EAP20 resulted in a substantial reduction in dsRNA signal intensity (Fig. 4A and B). Additionally, RT-qPCR results revealed a significant decrease in intracellular viral RNA copies at 10 hpi (Fig. 4C). Strand-specific RT-qPCR further revealed that *EAP20* knockdown impaired the synthesis of both genomic RNA (gRNA) and subgenomic RNAs (sgRNA2–7, Fig. 4D), confirming that EAP20 contributes to PRRSV RNA replication and transcription. To assess whether EAP20 affected viral assembly, the relative intracellular PRRSV-specific infectivity was determined by comparing the intracellular viral infectious titers with the intracellular viral RNA levels (28). Analysis of virus assembly showed a minor reduction in PRRSV particle production upon *EAP20* knockdown (Fig. 4E). Additionally, the ratio of intracellular and extracellular viral infectivity to total infectivity remained unchanged, indicating that EAP20 is dispensable for PRRSV release (Fig. 4F).

Given that EAP20 functions during the viral entry, we further bypassed this step by transfecting HEK-293T cells (non-susceptible to PRRSV) with an infectious PRRSV complementary (cDNA) clone (HN07-GFP) (29) to assess the impact of EAP20 on viral replication, assembly, and release. Under these conditions, *EAP20* silencing significantly inhibited PRRSV RNA synthesis, whereas it exerted a negligible effect on viral assembly and release (Fig. 4G through I). Taken together, these findings demonstrate that EAP20 is primarily involved in PRRSV entry and replication.

### EAP20 facilitates the transport of the internalized PRRSV particles to EEs through the CME pathway

**Fig 5:**
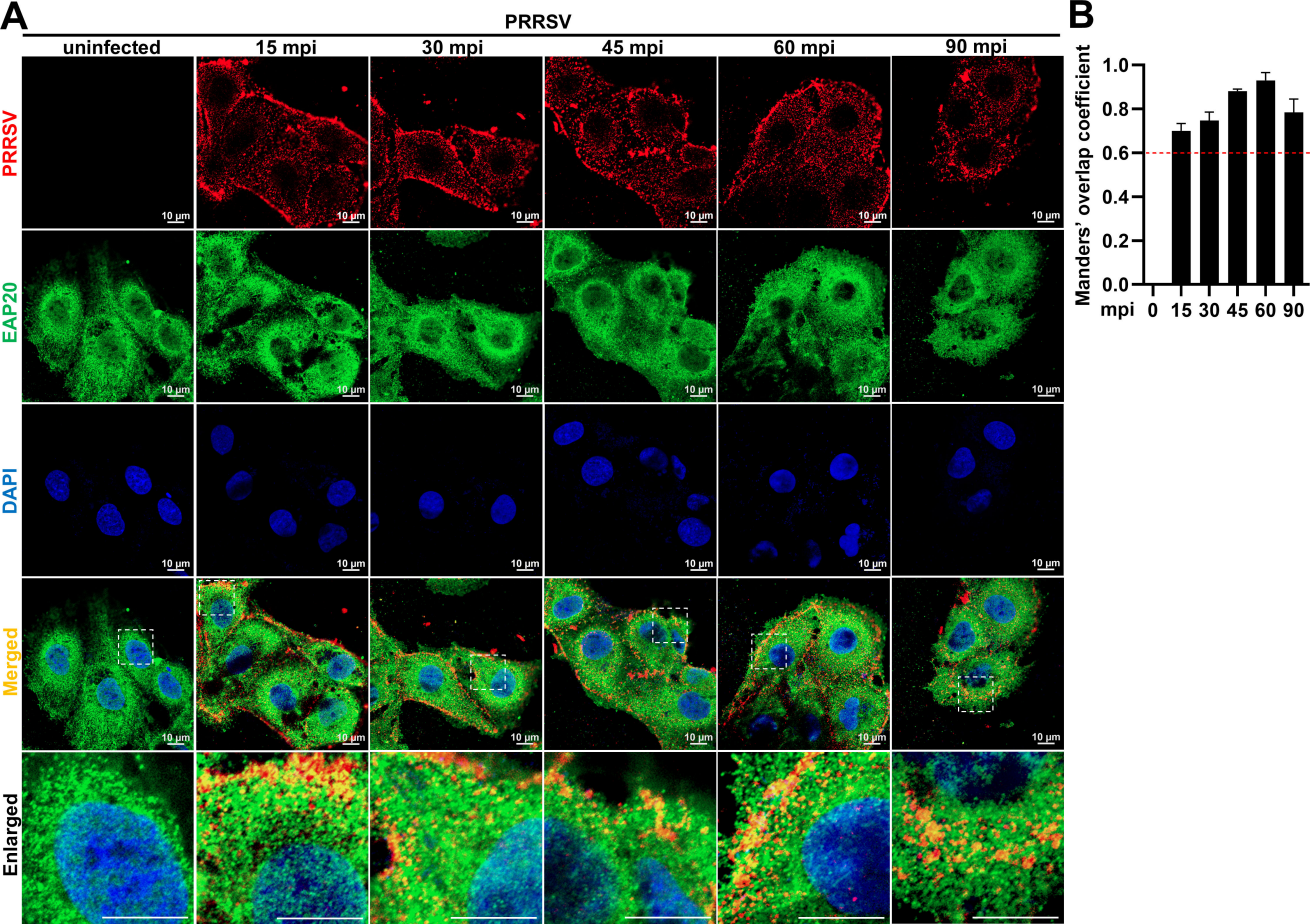
EAP20 colocalized with PRRSV at the internalization stage. (**A**) The MARC-145 cells were infected with or without 10 MOI PRRSV at 4°C for 1 h, followed by transfer to 37°C and harvested at the indicated time points (0, 15, 30, 45, 60, or 90 mpi). The cells were fixed, penetrated, blocked, and then incubated with mouse anti-PRRSV N MAb and rabbit anti-EAP20 pAbs, followed by incubation with Alexa Fluor 488-donkey anti-mouse IgG pAbs and Alexa Fluor 647-donkey anti-rabbit IgG pAbs. The fluorescence signals of PRRSV N protein (red) and EAP20 (green) were visualized and captured using confocal microscopy. Nuclei were stained blue with DAPI, and the scale bars were indicated as 10 μm. These images represent at least 10 analyzed cells from three independent experiments. (**B**) The colocalization between N protein and EAP20 was evaluated by determining the Manders’ overlap coefficient via the JaCoP plugin within the ImageJ software. Data are shown as means ± SEM from three individual enlarged images.

To further investigate the involvement of EAP20 in the viral entry, we first monitored the localization of endogenous EAP20 with PRRSV particles using confocal microscopy. Upon PRRSV infection, the cytoplasmic EAP20 progressively colocalized with PRRSV particles over time (Fig. 5A, the fourth row), as confirmed by quantitative Manders’ overlap coefficient analysis (>0.6; Fig. 5B). These results suggest that EAP20 accumulates at the PRRSV entry routes during early infection.

**Fig 6:**
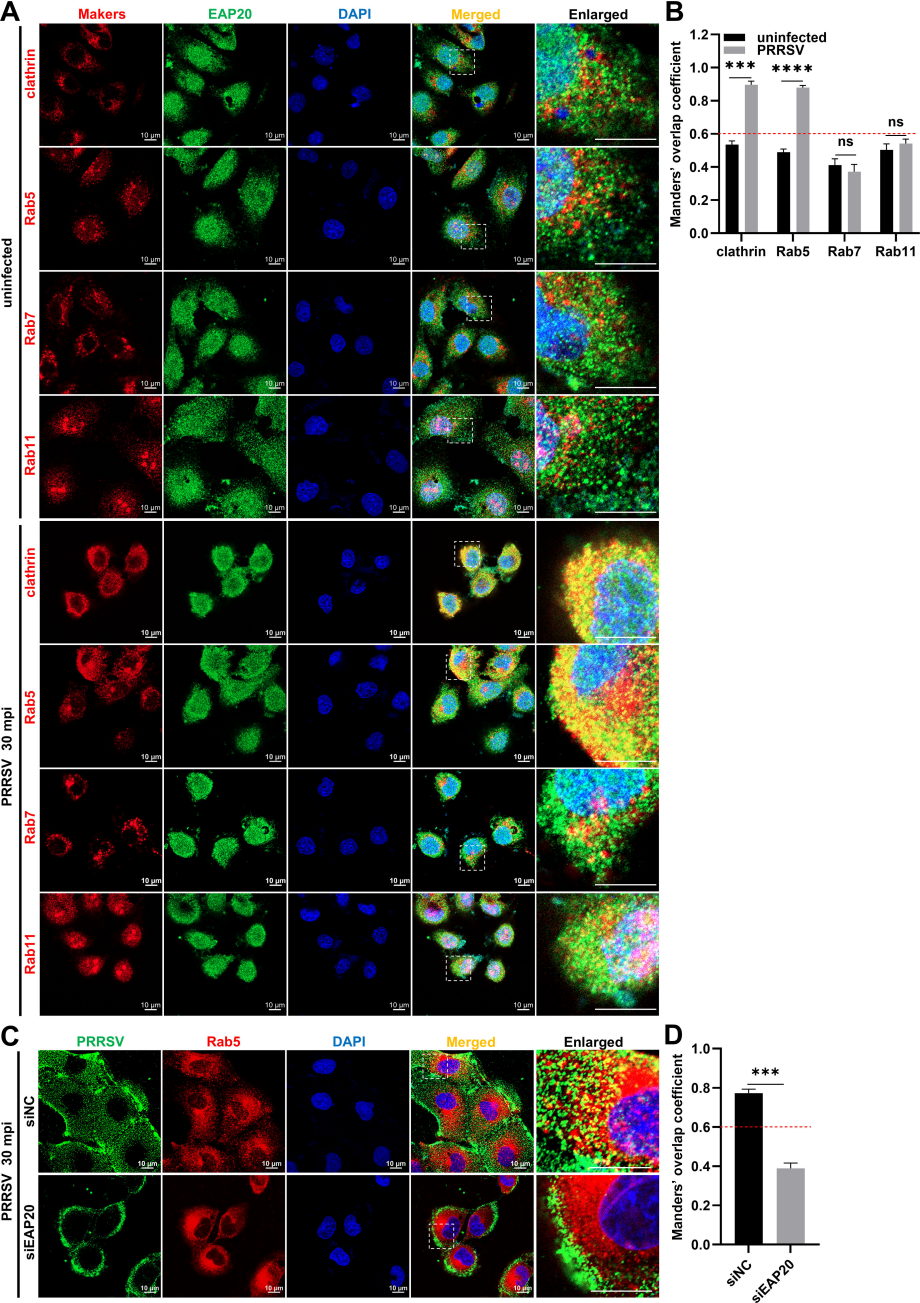
EAP20 facilitates the transport of the internalized PRRSV particles to EEs via the CME pathway. (**A**) MARC-145 cells were infected with or without PRRSV (MOI = 10) and incubated at 4°C for 1 h, followed by transfer to 37°C for 30 min. The cells were fixed and then co-incubated with mouse anti-EAP20 MAb and rabbit anti-clathrin, Rab5, Rab7, or Rab11 antibodies. After incubation with Alexa Fluor 488-donkey anti-mouse IgG pAbs and Alexa Fluor 647-donkey anti-rabbit IgG pAbs, confocal microscopy was used to visualize their subcellular localization. Nuclei were stained blue with DAPI, and the scale bars were indicated as 10 μm. These images represent at least 10 analyzed cells from three independent experiments. (**B**) The colocalization between EAP20 and the markers of distinct endocytic compartments was evaluated by determining the Manders’ overlap coefficient via the JaCoP plugin within the ImageJ software. Data are shown as means ± SEM from three individual enlarged images. ***, *P* < 0.001; ****, *P* < 0.0001. ns, not significant, *P* > 0.05. *P*-values were analyzed by unpaired two-tailed Student’s *t*-tests. (**C**) MARC-145 cells were transfected with siNC or siEAP20 for 36 h and then infected with PRRSV (MOI = 10) at 37°C for 30 min. The cells were fixed and then co-incubated with mouse anti-PRRSV N MAb and rabbit anti-Rab5 pAbs, followed by incubation with Alexa Fluor 647-donkey anti-rabbit IgG pAbs and Alexa Fluor 488-donkey anti-mouse IgG pAbs. Confocal microscopy was applied to observe their subcellular localization after the nuclei were dyed blue with DAPI. Scale bars were indicated as 10 μm. These images represent at least 10 analyzed cells from three independent experiments. (**D**) The colocalization between PRRSV and EEs was evaluated by determining the Manders’ overlap coefficient via the JaCoP plugin within the ImageJ software. Data are shown as means ± SEM from three individual enlarged images. ***, *P* < 0.001. *P*-values were analyzed by unpaired two-tailed Student’s *t*-tests.

Previous studies have demonstrated that PRRSV primarily enters the host cells through CME, with macropinocytosis serving as a secondary pathway (30–32). In the CME route, PRRSV virions are initially internalized into the clathrin-coated vesicles (CCVs) and then trafficked to EEs and recycling endosomes (REs), but not to late endosomes (LEs) (33). To explore whether EAP20 participated in PRRSV CME-dependent entry, we analyzed its colocalization with the markers of endocytic compartments, including clathrin (CCVs), Rab5 (EEs), Rab7 (LEs), and Rab11 (REs) in both the infected and uninfected cells. As shown in Fig. 6A and B, PRRSV proliferation significantly enhanced the colocalization of EAP20 with clathrin and Rab5, but not with Rab7 or Rab11. In contrast, *EAP20* knockdown led to a significant reduction in the number of PRRSV particles colocalized with EEs (Fig. 6C and D). These results indicate that EAP20 participates in the trafficking of internalized PRRSV particles to EEs via the CME pathway.

**Fig 7:**
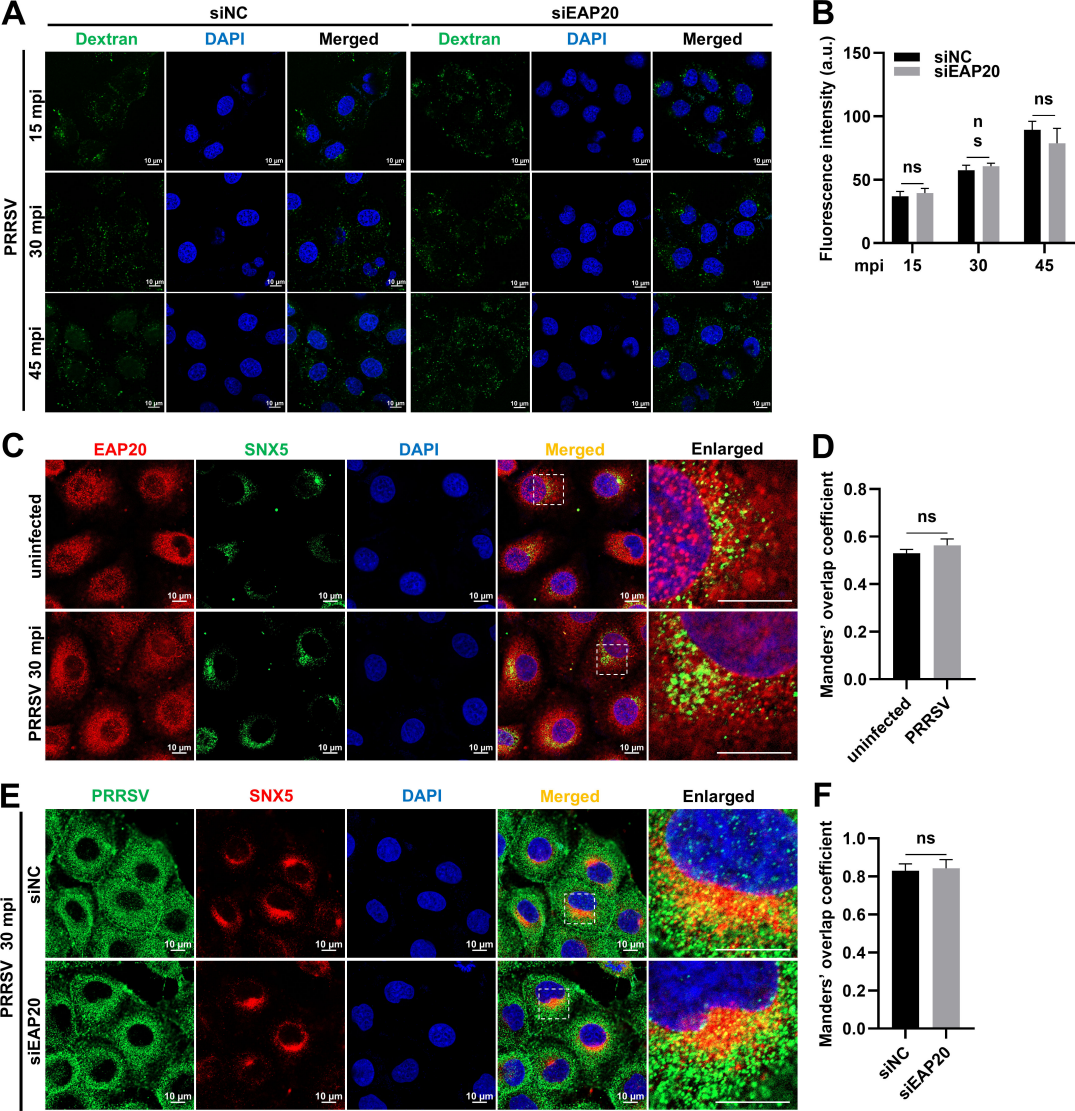
EAP20 is not required for PRRSV macropinocytosis-dependent entry. (**A**) Silencing EAP20 did not affect dextran uptake. The MARC-145 cells transfected with siNC or siEAP20 were serum-starved for 2 h. Then, the cells were inoculated with PRRSV at 10 MOI and incubated at 4°C for 1 h. After washing with PBS, the cells were cultured using DMEM containing a final concentration of 250 μg/mL fluorescein isothiocyanate (FITC)-conjugated dextran and transferred to 37°C for further incubation for 15, 30, or 45 min. After the cells were harvested and fixed, the images were observed and captured using confocal microscopy. These images represent at least 10 analyzed cells from three independent experiments. (**B**) The total green intensity represented the dextran uptake by cells and was statistically analyzed using the ImageJ software. Data are shown as means ± SEM from three individual images. ns, not significant, *P* > 0.05. *P*-values were analyzed by unpaired two-tailed Student’s *t*-tests. (**C**) Colocalization analysis between EAP20 and the macropinosome marker SNX5. The MARC-145 cells infected with or without PRRSV (MOI = 10) were harvested at 30 mpi. After fixation, the cells were incubated with mouse anti-EAP20 MAb and rabbit anti-SNX5 pAbs, followed by incubation with Alexa Fluor 488-donkey anti-rabbit IgG pAbs and Alexa Fluor 647-donkey anti-mouse IgG pAbs. Confocal microscopy was used to observe the subcellular localization of proteins. Nuclei were stained blue with DAPI, and the scale bars were indicated as 10 μm. These images represent at least 10 analyzed cells from three independent experiments. (**D**) The colocalization between EAP20 and SNX5 was evaluated by determining the Manders’ overlap coefficient via the JaCoP plugin within the ImageJ software. (**E**) Silencing EAP20 did not affect the colocalization of PRRSV and SNX5. The MARC-145 cells transfected with siNC or siEAP20 were infected with PRRSV (MOI = 10) at 37°C for 30 min. After fixation, the cells were incubated with mouse anti-PRRSV N MAb and rabbit anti-SNX5 pAbs, followed by incubation with Alexa Fluor 488-donkey anti-mouse IgG pAbs and Alexa Fluor 647-donkey anti-rabbit IgG pAbs. Nuclei were stained blue with DAPI, and the scale bars were indicated as 10 μm. These images represent at least 10 analyzed cells from three independent experiments. (**F**) The colocalization between PRRSV N and SNX5 was evaluated by determining the Manders’ overlap coefficient via the JaCoP plugin within the ImageJ software. Data are shown as means ± SEM from three individual enlarged images. ns, not significant, *P* > 0.05. *P*-values were analyzed by unpaired two-tailed Student’s *t*-tests.

We further explored whether EAP20 was involved in PRRSV macropinocytosis-dependent entry. For this purpose, we first assessed the uptake of dextran, a fluid-phase marker of macropinocytosis (34), by confocal microscopy at different time points post-infection. Total fluorescence intensity analysis revealed that dextran uptake began as early as 15 min post-infection (mpi) and increased over time, but *EAP20* knockdown had no significant effect on dextran internalization (Fig. 7A and B). Additionally, we employed confocal microscopy to visualize the localization of endogenous EAP20 with the sorting nexin 5 (SNX5), a well-established marker of macropinosomes (the specific endosomes for macropinocytosis) (35). Confocal imaging revealed that there was no notable colocalization between EAP20 and SNX5, regardless of PRRSV infection (Fig. 7C and D). Furthermore, our findings revealed a significant colocalization between PRRSV N and SNX5 during early infection, whereas *EAP20* knockdown did not alter this colocalization (Fig. 7E and F), suggesting that the recruitment of PRRSV to SNX5-positive compartments occurs independently of EAP20 function. Overall, these results indicate that EAP20 is not required for PRRSV macropinocytosis-dependent entry.

### EAP20 interacts with PRRSV Nsps

**Fig 8:**
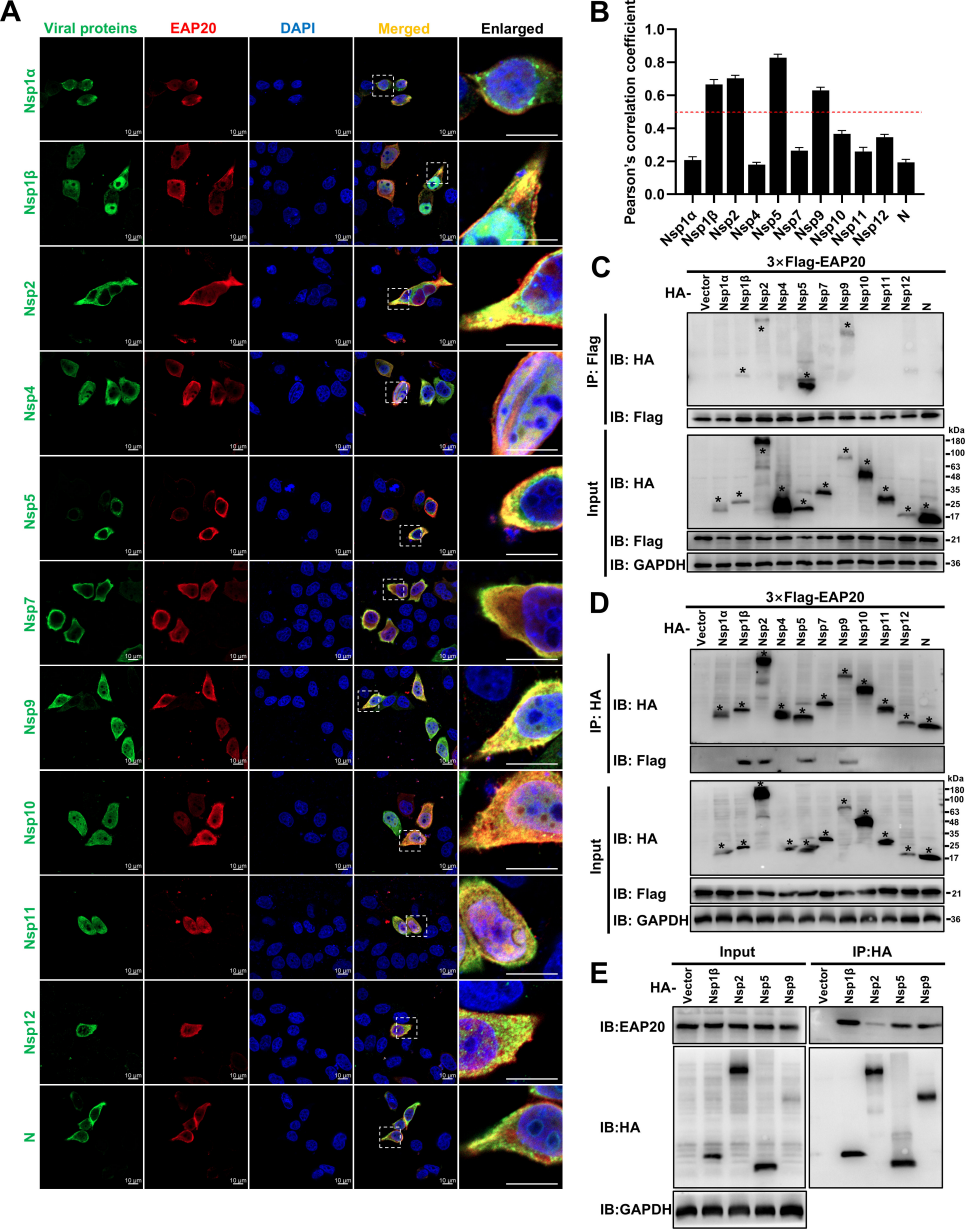
Interaction between EAP20 and PRRSV Nsps. (**A**) HeLa cells were co-transfected with 3 × Flag-EAP20 plasmids and HA-tagged PRRSV protein plasmids (HA-Nsp1α, -Nsp1β, -Nsp2, -Nsp4, -Nsp5, -Nsp7, -Nsp9-12, and -N) at 37°C for 36 h. After fixation, the cells were incubated with mouse anti-Flag MAb and rabbit anti-HA pAbs, followed by incubation with Alexa Fluor 488-donkey anti-rabbit IgG pAbs and Alexa Fluor 647-donkey anti-mouse IgG pAbs. Confocal microscopy was applied to observe their subcellular localization after the nuclei were dyed blue with DAPI. Scale bars were indicated as 10 μm. These images represent at least 10 analyzed cells from three independent experiments. (**B**) The colocalization between EAP20 and the indicated PRRSV proteins was evaluated by determining the Pearson’s correlation coefficient via the JaCoP plugin within the ImageJ software. Data are shown as means ± SEM from three individual enlarged images. (**C and D**) Interaction analysis of EAP20 and PRRSV proteins by Co-IP. HEK-293T cells were co-transfected with 3× Flag-EAP20 plasmids and HA-tagged PRRSV protein plasmids (HA-Nsp1α, -Nsp1β, -Nsp2, -Nsp4, -Nsp5, -Nsp7, -Nsp9-12, and -N) or HA-tagged empty vectors at 37°C for 36 h. After lysis and centrifugation, the obtained WCLs were immunoprecipitated with anti-Flag magnetic beads (**C**) or anti-HA magnetic beads (**D**) and then subjected to IB to identify the potential interacting proteins. Asterisks (*) denote the expressed HA-tagged PRRSV proteins on the membranes. (**E**) HEK-293T cells were transfected with the HA-tagged PRRSV protein plasmids (HA-Nsp1β, -Nsp2, -Nsp5, and -Nsp9) or HA-tagged empty vectors at 37°C for 36 h. After lysis and centrifugation, the obtained WCLs were immunoprecipitated with anti-HA magnetic beads, and then endogenous proteins were detected by IB using rabbit-anti EAP20 pAbs. GAPDH served as an internal control in the Input group.

To explore the potential mechanisms by which EAP20 promoted PRRSV replication, we investigated its interactions with viral proteins using confocal microscopy and co-immunoprecipitation (Co-IP) assays. Confocal microscopy revealed that EAP20 specifically colocalized with Nsp1β, Nsp2, Nsp5, and Nsp9 in the HeLa cells co-expressing Flag-tagged EAP20 and hemagglutinin (HA)-tagged PRRSV proteins (HA-Nsp1α, -Nsp1β, -Nsp2, -Nsp4, -Nsp5, -Nsp7, -Nsp9, -Nsp10, -Nsp11, -Nsp12, and -N) (Fig. 8A). Quantitative analysis using Pearson’s correlation coefficient confirmed these interactions (>0.5; Fig. 8B). These interactions were further validated by Co-IP in HEK-293T cells. As shown in Fig. 8C, Flag-EAP20 pulled down Nsp1β, Nsp2, Nsp5, and Nsp9 from whole-cell lysates (WCLs). In parallel, EAP20 was also specifically precipitated by these viral proteins (Fig. 8D). Furthermore, endogenous EAP20 interacted with HA-Nsp1β, -Nsp2, -Nsp5, and -Nsp9 (Fig. 8E). These results show that EAP20 interacts with a subset of PRRSV Nsps.

### EAP20 anchors Nsp9 on the perinuclear ER and coordinates with Nsp2/Nsp5 for DMV formation

**Fig 9:**
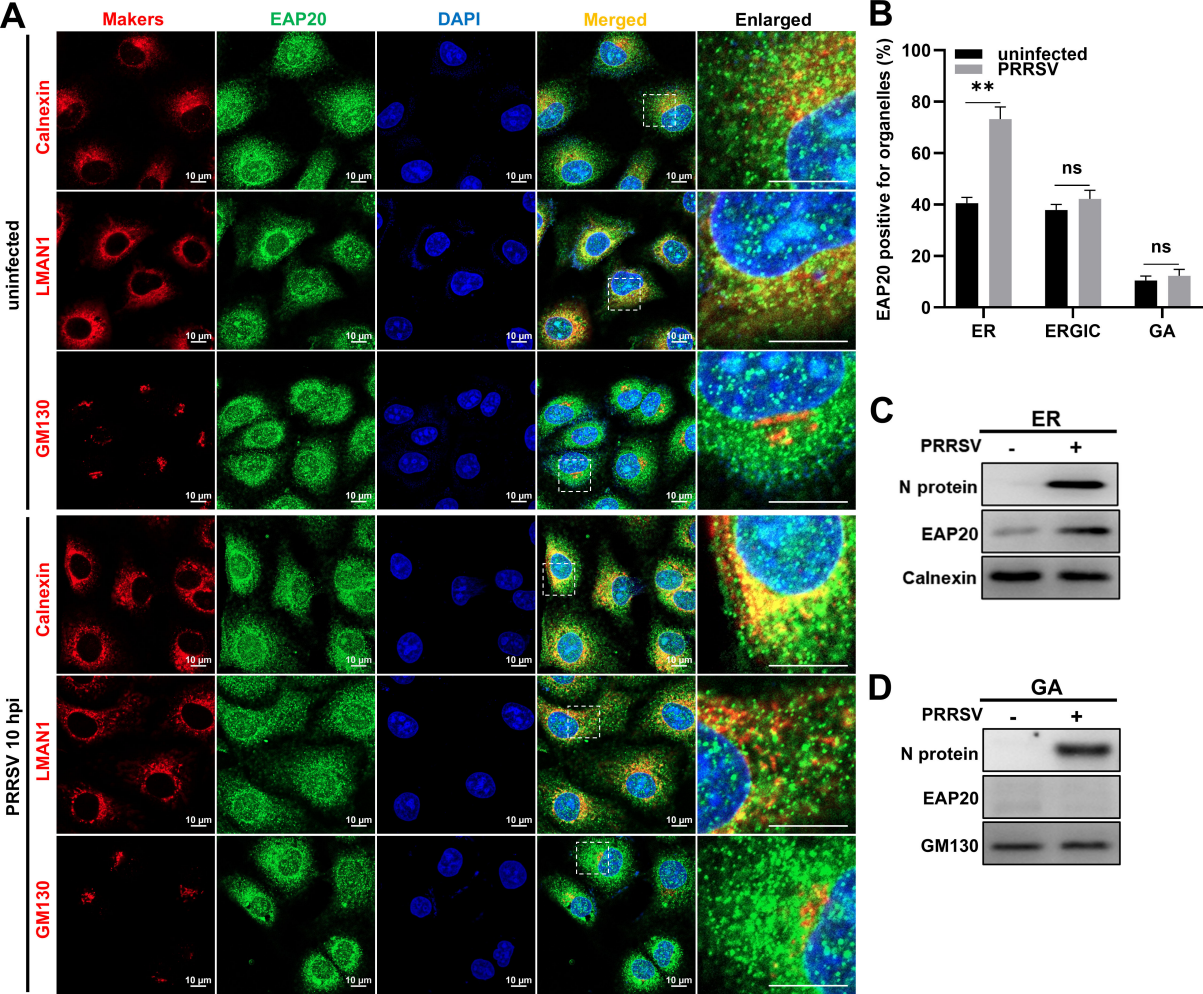
EAP20 localizes at the ER during PRRSV replication. (**A**) MARC-145 cells were infected with or without PRRSV (MOI = 10) at 37°C for 10 h. The cells were then co-incubated with mouse anti-EAP20 MAb and rabbit anti-Calnexin, LMAN1, or GM130 antibodies. After incubation with Alexa Fluor 488-donkey anti-mouse IgG pAbs and Alexa Fluor 647-donkey anti-rabbit IgG pAbs, confocal microscopy was applied to observe the intracellular distribution profiles of these proteins. Nuclei were stained blue with DAPI, and the scale bars were indicated as 10 μm. These images represent at least 10 analyzed cells from three independent experiments. (**B**) Colocalization analysis was performed using the JaCoP plugin within ImageJ software by calculating Manders’ colocalization coefficient. Data are shown as means ± SEM from three individual enlarged images. **, *P* < 0.01; ns, not significant, *P* > 0.05. *P*-values were analyzed by unpaired two-tailed Student’s *t*-tests. (**C and D**) The MARC-145 cells seeded in 100 mm dishes were infected with or without PRRSV (MOI = 10). After incubation at 37°C for 10 h, the cells were transferred to −80°C for freezing. The ER (**C**) and GA (**D**) were individually purified using a commercial Minute ER (or GA) enrichment kit and then subjected to IB to detect the levels of EAP20 and PRRSV N proteins in the enriched organelle fractions. Calnexin and GM130 served as loading controls and were also used to verify the purity of the organelle fractionation.

Given that Nsp1β, Nsp2, Nsp5, and Nsp9 are implicated in PRRSV replication on the perinuclear ER (27, 36, 37), we first investigated the subcellular localization of EAP20 with the membranous organelles ER, ER-Golgi intermediate compartment (ERGIC), and Golgi apparatus (GA) during this stage. As depicted in Fig. 9A, EAP20 colocalized with the ER marker Calnexin in the PRRSV-infected MARC-145 cells at 10 hpi, but not with the ERGIC marker LMAN1 or the GA marker GM130. Notably, the proportion of EAP20-positive ER remarkably increased from ~40% in the uninfected cells to ~75% following PRRSV infection (Fig. 9B). In parallel, we employed a commercial organelle isolation kit to quantify the organelle-localized EAP20 by IB, and the results showed that the abundance of EAP20 was increased in ER extracts, whereas it remained undetectable in GA fractions during PRRSV replication (Fig. 9C and D; middle panels). The purity of the isolated ER and GA fractions was verified by IB for the markers Calnexin and GM130, respectively. Minimal cross-contamination between the fractions was observed, confirming the fidelity of the fractionation procedure (Fig. 9C and D; bottom panels).

**Fig 10:**
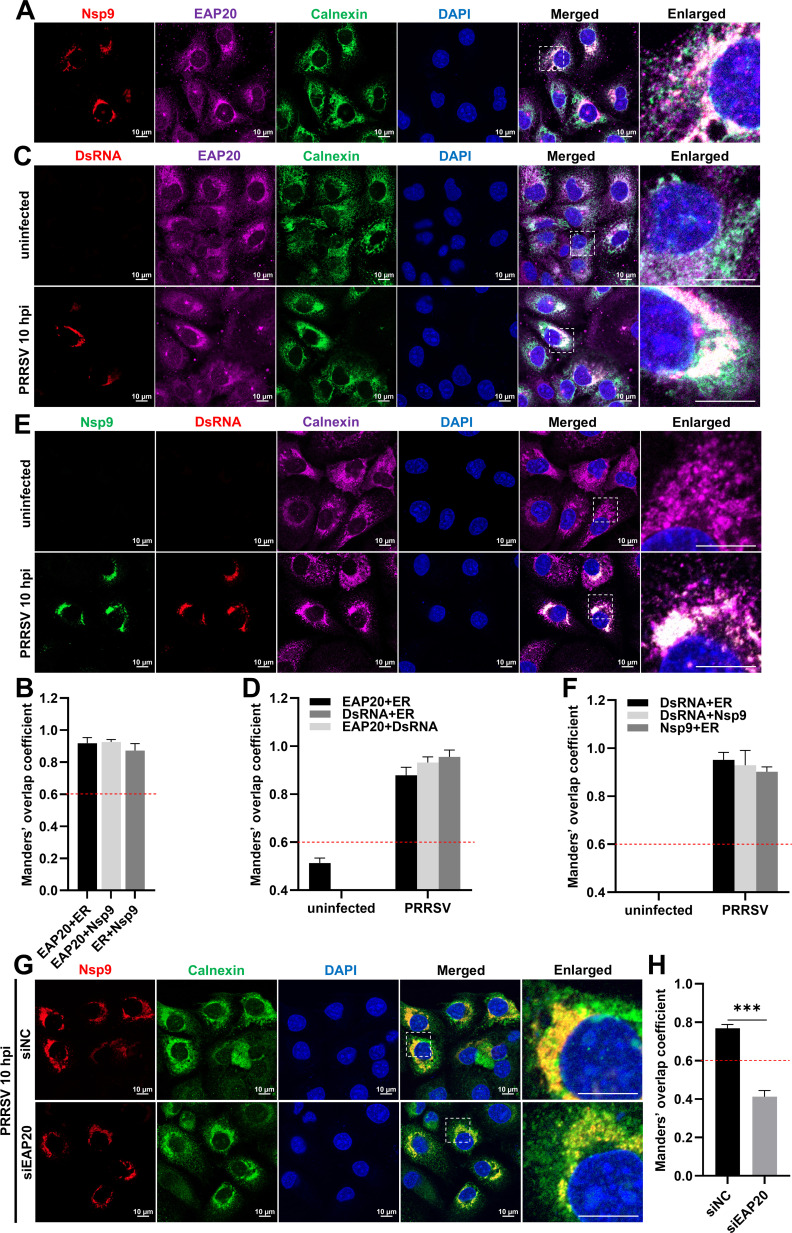
Colocalization analysis among PRRSV Nsp9, EAP20, and dsRNA on the ER. (**A**) MARC-145 cells were transfected with HA-Nsp9 at 37°C for 36 h. The cells were fixed and then co-incubated with rabbit anti-EAP20 pAbs, mouse anti-HA MAb, and goat anti-Calnexin pAbs, followed by incubation with Alexa Fluor 488-donkey anti-goat IgG pAbs, Alexa Fluor 555-donkey anti-rabbit IgG pAbs, and Alexa Fluor 647-donkey anti-mouse IgG pAbs. Confocal microscopy was applied to observe their subcellular localization after the nuclei were dyed blue with DAPI. Scale bars were indicated as 10 μm. These images represent at least 10 analyzed cells from three independent experiments. (**B**) The colocalization among PRRSV Nsp9, EAP20, and ER marker Calnexin was evaluated by determining the Manders’ overlap coefficient via the JaCoP plugin within the ImageJ software. Data are shown as means ± SEM from three individual enlarged images. (**C**) MARC-145 cells were infected with or without PRRSV (MOI = 10) at 37°C for 10 h. The cells were fixed and then co-incubated with mouse anti-dsRNA MAb, rabbit anti-EAP20, and goat anti-Calnexin pAbs, followed by incubation with the same fluorescent secondary antibodies as in (**A**). Confocal microscopy was applied to observe their subcellular localization after the nuclei were dyed blue with DAPI. Scale bars were indicated as 10 μm. These images represent at least 10 analyzed cells from three independent experiments. (**D**) The colocalization among PRRSV dsRNA, EAP20, and Calnexin was evaluated by determining the Manders’ overlap coefficient via the JaCoP plugin within the ImageJ software. Data are shown as means ± SEM from three individual enlarged images. (**E**) MARC-145 cells were subjected to similar treatments as in (**C**), whereas the fixed cells were co-incubated with mouse anti-dsRNA MAb, Nsp9 nanobody (Nb6-pFc), and rabbit anti-Calnexin pAbs, followed by incubation with FITC-goat anti-pig IgG pAbs, Alexa Fluor 555-donkey anti-rabbit IgG pAbs, and Alexa Fluor 647-donkey anti-mouse IgG pAbs. Nuclei were dyed blue with DAPI. The cells were visualized using confocal microscopy. Scale bars were indicated as 10 μm. These images represent at least 10 analyzed cells from three independent experiments. (**F**) The colocalization among PRRSV dsRNA, Nsp9, and Calnexin was expressed as Manders’ overlap coefficient. Data are shown as means ± SEM from three individual enlarged images. (**G**) MARC-145 cells were transfected with either siEAP20 or siNC for 6 h and then transfected with HA-Nsp9 for 24 h. The cells were harvested, fixed, and then co-incubated with mouse anti-HA MAb and rabbit anti-Calnexin pAbs, followed by incubation with Alexa Fluor 488-donkey anti-rabbit IgG pAbs and Alexa Fluor 647-donkey anti-mouse IgG pAbs. Confocal microscopy was used to observe its subcellular localization after the nuclei were dyed blue with DAPI. Scale bars were indicated as 10 μm. These images represent at least 10 analyzed cells from three independent experiments. (**H**) The colocalization of PRRSV Nsp9 with ER marker Calnexin was evaluated by determining the Manders’ overlap coefficient via the JaCoP plugin within the ImageJ software. Data are shown as means ± SEM from three individual enlarged images. ***, *P* < 0.001. *P*-values were analyzed by unpaired two-tailed Student’s *t*-tests.

As PRRSV Nsp9 is an integral replicase for the viral RNA synthesis (27, 36, 37), HA-Nsp9 was expressed in MARC-145 cells, and triple fluorescence confocal microscopy was conducted to further assess its relationship with EAP20. Figure 10A and B showed that HA-Nsp9 colocalized with endogenous EAP20 on the perinuclear ER. More importantly, endogenous EAP20, Nsp9, and dsRNA showed strong colocalization with each other on the perinuclear ER in the PRRSV-infected cells (Fig. 10C through F). In contrast, knockdown of EAP20 markedly attenuated the localization of Nsp9 on the ER (Fig. 10G and H). These results suggest that EAP20 interacts with PRRSV Nsp9 to anchor it on the perinuclear ER.

**Fig 11:**
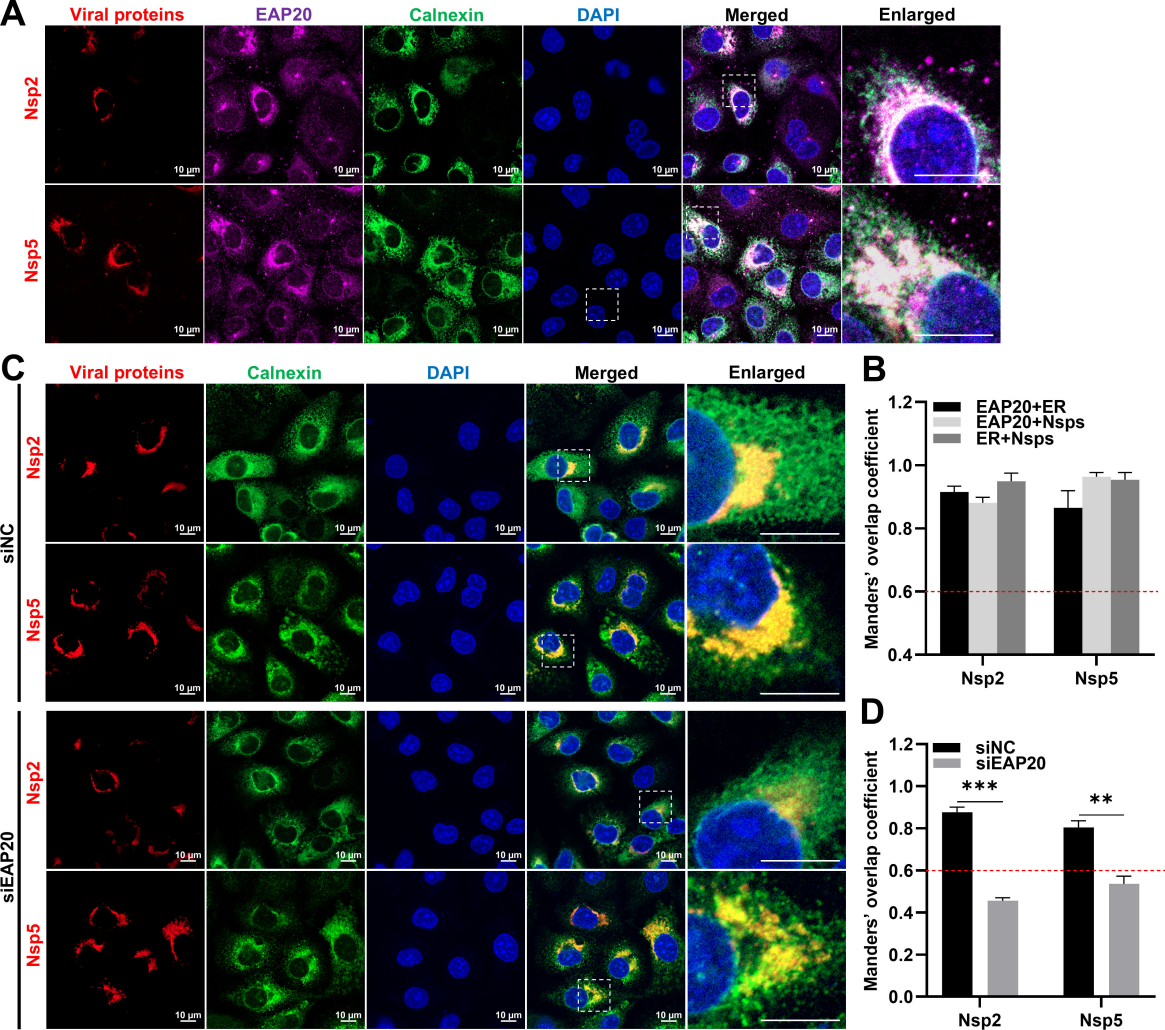
*EAP20* knockdown affects the subcellular localization of PRRSV Nsp2/Nsp5 with the ER. (**A**) MARC-145 cells were transfected with HA-Nsp2 and HA-Nsp5 at 37°C for 36 h. The cells were fixed and then co-incubated with rabbit anti-EAP20 pAbs, mouse anti-HA MAb, and goat anti-Calnexin pAbs, followed by incubation with Alexa Fluor 488-donkey anti-goat IgG pAbs, Alexa Fluor 555-donkey anti-rabbit IgG pAbs, and Alexa Fluor 647-donkey anti-mouse IgG pAbs. Confocal microscopy was applied to observe their subcellular localization after the nuclei were dyed blue with DAPI. Scale bars were indicated as 10 μm. These images represent at least 10 analyzed cells from three independent experiments. (**B**) The colocalization among PRRSV Nsp2/Nsp5, EAP20, and ER marker Calnexin was evaluated by determining the Manders’ overlap coefficient via the JaCoP plugin within the ImageJ software. Data are shown as means ± SEM from three individual enlarged images. (**C**) MARC-145 cells were transfected with either siEAP20 or siNC for 6 h and then transfected with HA-Nsp2 and HA-Nsp5 for 24 h. The cells were harvested, fixed, and then co-incubated with mouse anti-HA MAb and rabbit anti-Calnexin pAbs, followed by incubation with Alexa Fluor 488-donkey anti-rabbit IgG pAbs and Alexa Fluor 647-donkey anti-mouse IgG pAbs. Confocal microscopy was used to observe its subcellular localization after the nuclei were dyed blue with DAPI. Scale bars were indicated as 10 μm. These images represent at least 10 analyzed cells from three independent experiments. (**D**) The colocalization of PRRSV Nsp2/Nsp5 with ER marker Calnexin was evaluated by determining the Manders’ overlap coefficient via the JaCoP plugin within the ImageJ software. Data are shown as means ± SEM from three individual enlarged images. **, *P* < 0.01; ***, *P* < 0.001. *P*-values were calculated using unpaired two-tailed Student’s *t*-tests.

**Fig 12:**
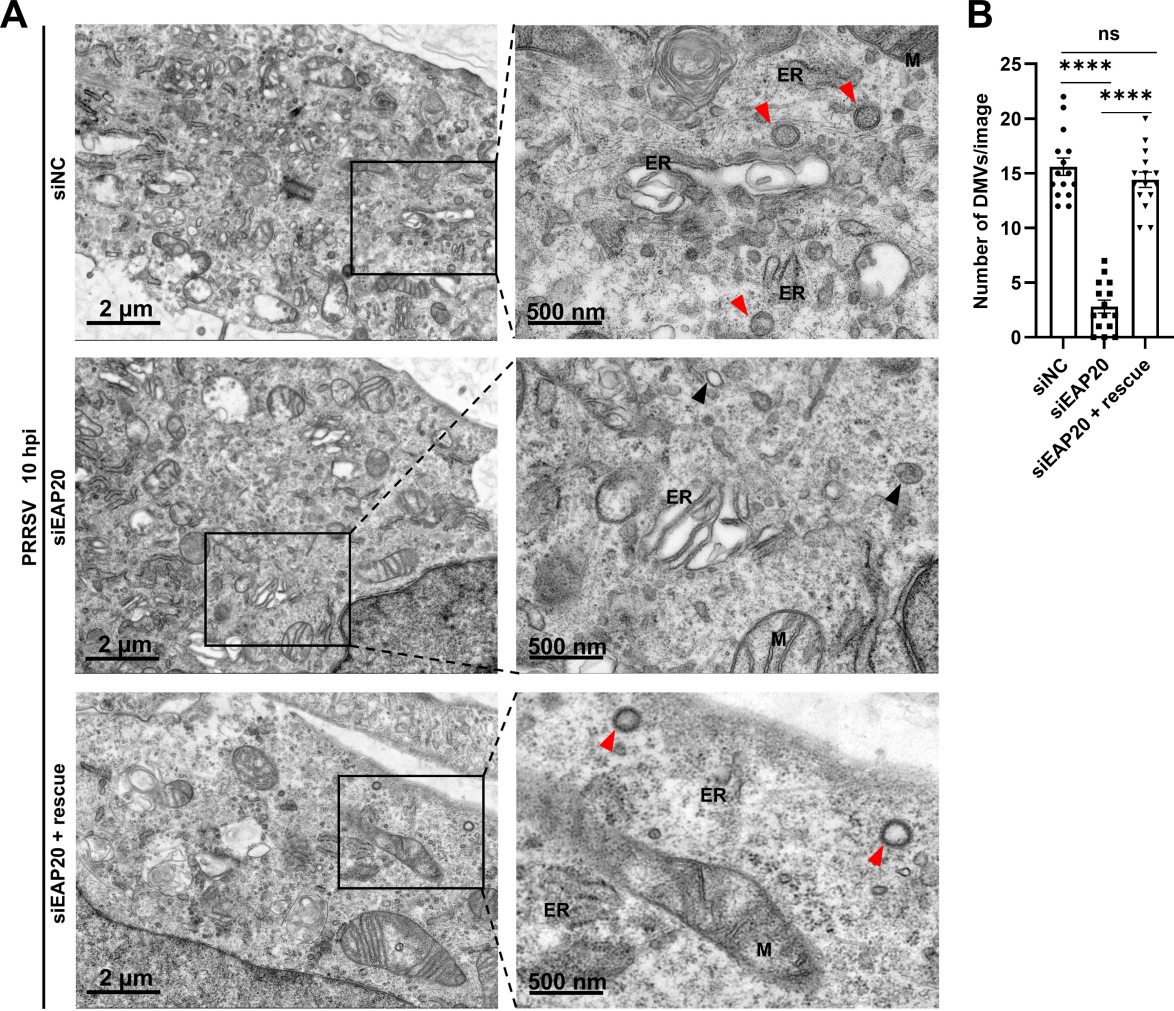
EAP20 contributes to PRRSV-induced DMV formation. (**A**) The MARC-145 cells seeded in 60 mm dishes were transfected with siNC, siEAP20, or siEAP20, followed by transfection with a plasmid harboring synonymous mutations in the EAP20 silencing sites (named as rescue) for 36 h and then infected with PRRSV (MOI = 10) at 37°C for 10 h. TEM was used to evaluate the morphological alterations of PRRSV-induced DMVs. The right panels displayed magnified views of the boxed regions. Red arrowheads represented the typical DMVs. Black arrowheads represented the DMVs with irregular morphology. M, mitochondria; ER, endoplasmic reticulum. Scale bars for low and high magnification were indicated as 2 μm and 500 nm, respectively. (**B**) The number of typical DMVs induced by PRRSV in each low magnification image was counted and shown as the means ± SEM (*n* = 15) from three independent experiments. ****, *P* < 0.0001. ns, not significant, *P* >0.05. *P*-values were analyzed by unpaired two-tailed Student’s *t*-tests.

Arteriviruses have been shown to hijack and remodel host ER membrane to form DMVs via the transmembrane proteins Nsp2, Nsp3, and Nsp5, providing a favorable microenvironment for viral RNA replication (27, 38–41). Here, we identified that EAP20 colocalized with Nsp2 and Nsp5 on the perinuclear ER using confocal microscopy (Fig. 11A and B). However, we observed a significant reduction in the localization of Nsp2/Nsp5 on the ER upon *EAP20* knockdown (Fig. 11C and D). We further examined the role of EAP20 on DMV formation by transmission electron microscopy (TEM). In control cells, we observed numerous typical DMVs (with a diameter of approximately 100 nm) containing small amounts of dense fibrous materials (Fig. 12A, top row), consistent with a previous report (41). In contrast, *EAP20* knockdown cells exhibited fewer DMVs, most of which displayed irregular morphology (Fig. 12A, middle row). Importantly, transfection of a siEAP20-resistant construct restored the formation of DMVs to the control levels (Fig. 12A, bottom row). Quantitative analysis across 15 low-magnification images randomly selected from three different experiments showed that *EAP20* knockdown reduced the number of typical DMVs by ~7-fold, while EAP20 complementation rescued this reduction (Fig. 12B). These findings demonstrate that EAP20 interacts with Nsp2 and Nsp5 on the perinuclear ER for DMV formation.

## DISCUSSION

It is well known that the ESCRT system regulates endosomal trafficking, organelle homeostasis, multivesicular body formation, autophagosome closure, and microvesicle biogenesis and secretion (16–18, 42, 43). Various viruses have evolved to hijack the host ESCRT machinery to benefit their life cycle during infection (44, 45). Our previous study has unveiled that some ESCRT proteins are involved in PRRSV proliferation (25), but the underlying mechanisms remain to be further elucidated. In the present work, we elucidated that PRRSV selectively exploited EAP20, a core subunit of ESCRT-II, to participate in viral entry and replication.

We initially identified that depletion of EAP20 markedly impaired the proliferation of multiple PRRSV strains across different cell types (Fig. 1), while the depletion of its partners, EAP30 and EAP45, did not yield similar effects. This subunit-specific dependency aligns with previous evidence that different ESCRT-II components play nonredundant roles in viral proliferation. For instance, EAP20 is exclusively required by avian sarcoma virus for the release of its Gag proteins (46). The selective engagement of EAP20 by PRRSV suggests that this subunit performs unique molecular functions that cannot be substituted by other ESCRT-II components. Our knockdown and overexpression experiments revealed that EAP20 was required for PRRSV proliferation and that its expression is neither transcriptionally nor translationally regulated during the infection process (Fig. 2). This mode of action is similar to the findings in classical swine fever virus (CSFV), which also employs EAP20 to enhance viral proliferation without modulating its expression level (47).

Stage-specific assays further established that EAP20 acted on PRRSV entry and replication (Fig. 3 and 4). Mechanistically, EAP20 facilitated endocytic trafficking of PRRSV particles to EEs via the CME pathway (Fig. 5 to 7). Several ESCRT components, including EAP20, are shown to facilitate the entry of certain enveloped and non-enveloped viruses, such as rotavirus (20), echovirus 1 (48), Crimean-Congo hemorrhagic fever virus (49), CSFV (47, 50), porcine epidemic diarrhea virus (PEDV), and porcine enteric alphacoronavirus (PEAV) (51). Given the inherent function of the ESCRT machinery in endocytosis and cargo transport (16, 17, 52), our findings suggest that EAP20 acts as a molecular adaptor that links the PRRSV entry process to the host endosomal trafficking machinery.

Beyond its role in viral entry, EAP20 also contributed to PRRSV replication. Our data indicated that EAP20 interacts with essential PRRSV Nsp1β, Nsp2, Nsp5, and Nsp9 (Fig. 8), each of which plays a distinct yet crucial role in viral PRRSV RNA synthesis (36, 37, 53–55). The functional relevance of these interactions is multifaceted and likely reflects EAP20’s coordination of different aspects of the replication process. Nsp9, as the core RNA-dependent RNA polymerase (RdRp), is central to the viral replication machinery (7). Our data demonstrate that EAP20 anchors Nsp9 to the perinuclear ER (Fig. 10), suggesting a critical role in stabilizing the replicase at the appropriate subcellular sites for efficient RNA synthesis. Nsp2 and Nsp5 are transmembrane proteins, and their interaction with EAP20 indicates that EAP20 assists in viral replication. Notably, Nsp1β is a key modulator of host innate immune responses, suppressing interferon production and host protein synthesis (56). The interaction between EAP20 and Nsp1β raises the intriguing possibility that EAP20 may also participate in viral immune evasion, perhaps by facilitating the proper localization or function of Nsp1β, a hypothesis that merits further investigation.

Why does EAP20 interact preferentially with this specific subset of Nsps? One possibility is that these Nsps collectively form the core of the RTC and/or are central to the establishment of the replication organelles. EAP20, as an ESCRT-II component with inherent membrane-binding and remodeling capabilities, could be strategically recruited to this complex to facilitate its assembly and membrane association. It remains an open question whether the interactions we observed are direct or mediated by other host or viral factors, which will be an important direction for future investigation.

During replication, EAP20 was specifically enriched on the ER, but not on the ERGIC or GA (Fig. 9). The mechanism driving this selective ER recruitment, whether through the recognition of viral proteins, sensing of membrane curvature induced by RTC, or interaction with other ER-localized host factors, remains to be fully elucidated. Importantly, EAP20 colocalized with both dsRNA and Nsp9 on the perinuclear ER, and its depletion markedly diminished the localization of Nsp9 to this compartment (Fig. 10). These observations support the model that EAP20 anchors the viral replicases to the ER, thereby facilitating RNA synthesis. Similarly, EAP20 interacted with Nsp2/Nsp5 on the perinuclear ER and was essential for DMV formation (Fig. 11 and 12), underscoring its role in organizing the membrane platform for viral replication.

Viral RNA synthesis within host cells is orchestrated by a complicated network of viral Nsps, dsRNA, and cellular factors, which is a rate-limiting step for viral proliferation (57). Arteriviruses-encoded transmembrane proteins Nsp2, Nsp3, and Nsp5 are known to induce ER membrane rearrangement and generate DMVs, which serve as membrane anchor platforms to further recruit viral replicase proteins together with cellular factors into their lumens for viral genome replication (27, 39, 58, 59). DMVs also provide a suitable microenvironment, which protects viral RNA from host nucleases and immune surveillance (40). The ESCRT system manipulates a variety of membrane remodeling events, including invagination and vesicle scission, exhibiting topological structural patterns similar to those observed during DMV formation (17). Therefore, we speculate that EAP20 may act alone or as an adaptor to recruit other ESCRT proteins and interact with PRRSV Nsp2/Nsp5 to induce DMV formation. Similar to our hypothesis, several ESCRT proteins have been shown to induce ER membrane rearrangement through interactions with the transmembrane proteins Nsp3/Nsp4 of PEDV, PEAV, and severe acute respiratory syndrome coronavirus 2, thereby facilitating DMV formation (51, 60). In addition, previous studies have revealed that EAP20 or other ESCRT components orchestrate with CSFV Nsps and dsRNA on the ER to form a functional replication-transcription complex (RTC) for viral genome synthesis (47, 50). Future work mapping the specific EAP20-binding domains within key Nsps like Nsp9 and assessing the virological consequences of disrupting these interactions will be crucial for understanding the precise mechanistic contribution of EAP20 to RTC assembly and function in PRRSV infection.

**Fig 13:**
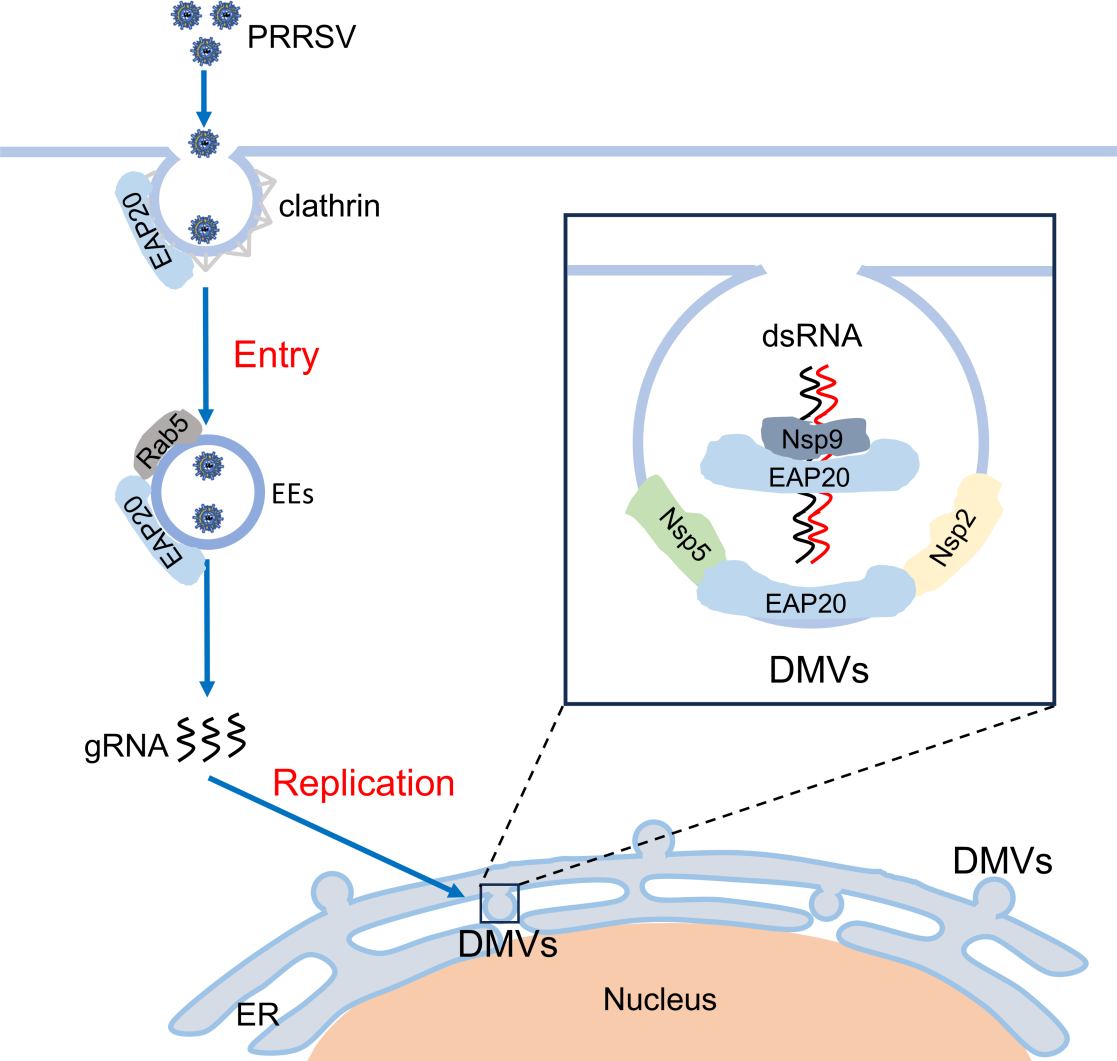
Schematic model illustrating the roles of EAP20 in the PRRSV life cycle. EAP20 contributes to PRRSV internalization and replication. Internalization stage: EAP20 transports the internalized PRRSV virions to EEs via the CME pathway. Replication stage: EAP20 interacts with Nsp2/Nsp5 for DMV formation, and it interacts and anchors Nsp9 on the perinuclear ER for viral RNA synthesis.

Based on the findings stated above, we present a dual-functional model in which EAP20 facilitates both PRRSV entry and replication (Fig. 13). During entry, EAP20 participates in the transport of internalized PRRSV virions to EEs via the CME pathway. During replication, EAP20 associates with PRRSV Nsps on the ER. Specifically, EAP20 anchors Nsp9 to the perinuclear ER and cooperates with Nsp2/Nsp5 to promote DMV formation for viral RNA synthesis. The interaction with Nsp1β further suggests a potential, yet to be defined, role in modulating host responses.

In summary, this study underlines a previously unrecognized function of the ESCRT-II subunit EAP20 in the PRRSV life cycle. The multifaceted roles of EAP20 highlight it as a pivotal host factor that bridges endocytic transport and membrane remodeling processes, both of which are essential for PRRSV proliferation. The specific interactions with key viral Nsps position EAP20 as a central coordinator of the viral replication organelle. These findings enrich our understanding of arterivirus-host interactions and identify EAP20 as a potential target for the development of antiviral strategies.

## MATERIALS AND METHODS

### Cells and viruses

MARC-145, HEK-293T, CRL-2843-CD163, and HeLa cell lines were all maintained in our laboratory (61). MARC-145, HEK-293T, and HeLa cells were cultured in Dulbecco’s modified Eagle’s medium (DMEM; #12100; Solarbio) supplemented with 10% heat-inactivated fetal bovine serum (FBS; #10270-106; Gibco) and antibiotics (100 U/mL penicillin, 100 μg/mL streptomycin; #P1400; Solarbio) in a humidified 37°C, 5% CO_2_ incubator. CRL-2843-CD163 cells were maintained in Roswell Park Memorial Institute 1640 medium (RPMI 1640; #31800; Solarbio) with 10% FBS and antibiotics under identical conditions.

Highly pathogenic (HP)-PRRSV strain HN07-1 (GenBank: KX766378.1) and HP-PRRSV strain HNhx (GenBank: KX766379; NADC30-like) were previously isolated in our laboratory (62, 63). Low pathogenic PRRSV strain BJ-4 (GenBank: AF331831) was kindly provided by Professor Hanchun Yang from China Agricultural University. The infectious clone of HN07-1 (HN07-GFP) was constructed and stored in our laboratory (29). The PRRSV strain HN07-1 was used in the experiments unless otherwise stated.

### Antibodies

Rabbit anti-clathrin polyclonal antibodies (pAbs, #2410S), rabbit anti-Rab5 monoclonal antibody (MAb, #3547S), rabbit anti-Rab7 MAb (#9367S), and rabbit anti-Rab11 MAb (#5589S) were purchased from Cell Signaling Technology. Mouse anti-glyceraldehyde-3-phosphate dehydrogenase (GAPDH) MAb (#60004-1-Ig) and rabbit anti-VPS25/EAP20 pAbs (#15669-1-AP) were purchased from Proteintech. Mouse anti-VPS25/EAP20 MAb (#sc-271648) was purchased from Santa Cruz. Rabbit anti-LMAN1 pAbs (#bs-18304R) were purchased from Bioss. Rabbit anti-SNX5 MAb (#ab180520), rabbit anti-Calnexin pAbs (#ab22595), goat anti-Calnexin pAbs (#ab219644), rabbit anti-GM130 MAb (#ab52649), Alexa Fluor 488-donkey anti-goat IgG pAbs (#150129), fluorescein isothiocyanate (FITC)-goat anti-pig IgG pAbs (#ab6911), and Alexa Fluor 555-donkey anti-rabbit IgG pAbs (#ab150074) were purchased from Abcam. FITC-conjugated dextran (#46945) was purchased from Sigma-Aldrich. Rabbit anti-PRRSV N pAbs (#GTX129270) were purchased from GeneTex. Mouse anti-dsRNA (J2) MAb (#10010200) was purchased from Scicons. Horseradish peroxidase (HRP)-goat anti-mouse IgG pAbs (#115-035-003) and HRP-goat anti-rabbit IgG pAbs (#111-035-003) were purchased from Jackson Immuno Research. Alexa Fluor 488-donkey anti-rabbit (#A-21206) or -mouse (#A-21202) IgG pAbs and Alexa Fluor 647-donkey anti-rabbit (#A-31573) or -mouse (#A-31571) IgG pAbs were purchased from Invitrogen. Mouse anti-HA MAb (#M2003M), rabbit anti-HA pAbs (#TT0050M), mouse anti-Flag MAb (#M2008M), anti-Flag magnetic beads (#M20118M), and anti-HA magnetic beads (#M20034M) were purchased from Ab-mart. Mouse anti-PRRSV N and GP5 MAb were prepared in our laboratory (32). PRRSV Nsp9 nanobody containing porcine IgG Fc (named as Nb6-pFc) was generously provided by Professor Qin Zhao from Northwest A&F University (64).

### Reagents

Phenylmethanesulfonyl fluoride (PMSF, #P0100), 4’,6’-diamidino-2-phenylindole (DAPI, #C0065), phosphate-buffered solution (PBS, #P1010), and 0.25% trypsin-EDTA solution (#T1300) were purchased from Solarbio. BeyoMag protein G plus magnetic beads (#P2106), Enhanced Cell Counting Kit-8 (CCK-8, #C0042), 4% paraformaldehyde (PFA, #P0099), Triton X-100 (#P0096), radioimmunoprecipitation assay (RIPA) lysis buffer (#P0013B), WB/IP lysis buffer (#P0013), BeyoECL Plus (#P0018S), and proteinase K (#ST535) were purchased from Beyotime Biotechnology. AceQ universal SYBR qPCR master mix (#Q511-03) was purchased from Vazyme Biotech. RNAiso Plus (#9109) and PrimeScript RT master mix (#RR036A) were purchased from TaKaRa. Complete EDTA-free protease inhibitor cocktail (#04693116001) was purchased from Roche. Lipofectamine RNAiMAX transfection reagent (#13778150), Lipofectamine LTX with Plus reagent (#15338030), and Lipofectamine 2000 reagent (#2066194) were purchased from Invitrogen. Minute ER enrichment kit (#ER-036) and Minute GA enrichment kit (#GO-037) were purchased from Invent Biotechnologies.

### Expression vector construction and transfection

The cDNA of *EAP20* (GenBank: XM_008012590) was amplified from MARC-145 cells and cloned into the p3 × Flag-CMV-7.1 plasmid to generate a recombinant p3 × Flag-EAP20 expression vector. The coding sequences of various PRRSV strain HN07-1 proteins were synthesized and cloned into the pCAGGS-HA plasmid by Sangon Biotech as previously described (25, 61). These plasmids were individually transfected into the indicated cells with Lipofectamine 2000 or Lipofectamine LTX with Plus reagent according to the manufacturer’s instructions.

### RNA interference assay

siRNA-NC and siRNAs targeting the ESCRT-II subunits (*EAP20*/*EAP30*/*EAP45*) were designed and synthesized by GenePharma. The MARC-145, CRL-2843-CD163, or HEK-293T cells in logarithmic growth phase were transfected with the indicated siRNAs at a final concentration of 50 or 100 nM for 36 h using Lipofectamine RNAiMAX according to the manufacturer’s instructions. The efficiencies of RNA silencing were determined at 36 hpt by RT-qPCR and IB. The viability of the transfected cells was confirmed using the CCK-8 assay according to the manufacturer’s instructions. The specific siRNA sequences are listed in Table 1.

**TABLE 1:** siRNA duplexes used in this study

Target gene	5’−3’ (sense)	5’−3’ (antisense)
EAP20 (monkey)	GAGUCAAUCCAGAUUGUAUTT	AUACAAUCUGGAUUGACUCTT
EAP30 (monkey)	UCUGAUAACUUUGGAGGAATT	UUCCUCCAAAGUUAUCAGATT
EAP45 (monkey)	CCGUUCUCCUUUCCCAAAUTT	AUUUGGGAAAGGAGAACGGTT
EAP20 (pig)	CGAUCCAGAUUGUAUUAGATT	UCUAAUACAAUCUGGAUCGTT
EAP30 (pig)	CUUGCAGAGGCCAAGUAUATT	UAUACUUGGCCUCUGCAAGTT
EAP45 (pig)	GCUGGAGGAAUAGGAAAGATT	UCUUUCCUAUUCCUCCAGCTT
Negative control	UUCUCCGAACGUGUCACGUTT	ACGUGACACGUUCGGAGAATT

### RT-qPCR

Total RNAs were extracted from the indicated cells using RNAiso Plus reagent, followed by reverse transcription using PrimeScript RT master mix kit. The cDNAs were amplified and monitored using AceQ universal SYBR qPCR master mix on a 7500 Fast instrument (Applied Biosystems). PRRSV RNA abundance was quantified with the primers targeting ORF7, and GAPDH served as an internal normalization control. The relative expression levels of target genes were calculated using the 2^-ΔΔCT^ method (65). All primer sequences used for RT-qPCR are listed in Table 2.

**TABLE 2:** Primers for RT-qPCR used in this study

Target gene	Sequence (5’−3’)	Usage
Leader-F	CACCTTGCTTCCGGAGTTG	gRNA and sgRNA2-7
sgRNA1-R	GAGAGACCGTGCACTGAGACATC	gRNA
sgRNA2-R	CAGCCAACCGGCGATTGTGAA	sgRNA 2
sgRNA3-R	GCAAAGCGGGCATACCGTGT	sgRNA 3
sgRNA4-R	ACGAAGTCTGATGCTGCGGTG	sgRNA 4
sgRNA5-R	CTGGCGTTGACGAGCACAGCA	sgRNA 5
sgRNA6-R	CATCACTGGCGTGTAGGTAATGGA	sgRNA 6
sgRNA7-F	CCCGGGTTGAAAAGCCTCGTGT	Total viral RNA
sgRNA7-R	GGCTTCTCCGGGTTTTTCTTCCTA	sgRNA 7 and total viral RNA
EAP20-F (monkey)	TGCTCGCTGGTCCTGTCCTT	Detection of EAP20 mRNAs
EAP20-R (monkey)	CCCTTTCTTCCTCAGTTCCTCTAA	
GAPDH-F (monkey)	TGACAACAGCCTCAAGATCG	Internal control
GAPDH-R (monkey)	GTCTTCTGGGTGGCAGTGAT	
GAPDH-F (pig)	CCTTCCGTGTCCCTACTGCCAAC	Internal control
GAPDH-R (pig)	GACGCCTGCTTCACCACCTTCT	

### Virus titration assay

The PRRSV-infected cells were harvested after washing with PBS at the indicated time points and underwent three freeze-thaw cycles at −80℃. The samples were centrifuged at 8,000 × *g* for 10 min to remove cellular debris. The resulting supernatants, containing intracellular infectious virions, were then titrated on MARC-145 cells to determine the TCID₅₀. The supernatants from the infected cells were collected for the measurement of extracellular virion titers. The PRRSV-containing solutions were diluted in a 10-fold serial gradient (10^−1^ to 10^−8^) with DMEM and then inoculated onto MARC-145 cells in 96-well plates. The cytopathic effect was monitored to calculate the TCID_50_ value according to the Reed-Muench method (66).

### IB

The cells seeded in 12-well plates were washed three times with pre-chilled PBS and lysed on ice for at least 30 min with the RIPA lysis buffer (100 to 300 μL) containing the 1× EDTA-free protease inhibitor cocktail and PMSF. Cell lysates were centrifuged at 12,000 × *g* at 4°C for 15 min; the resulting supernatants were denatured using SDS loading buffer by heating at 95°C for 10 min. Protein concentrations were standardized to GAPDH, separated through 10%–15% gradient SDS-PAGE, and transferred onto 0.22 µm PVDF membranes (#ISEQ00010, Merck Millipore). The membranes were then blocked with 5% skimmed milk in Tris-buffered saline containing 0.5% Tween 20 (TBST) at room temperature (RT) for 2 h and probed with the primary antibodies at 4°C overnight in NCM Universal Diluent (#WB500D, NCM Biotech). Afterward, the membranes were treated with the HRP-labeled secondary antibodies at RT for 1 h. Finally, protein bands were developed using BeyoECL Plus reagent, followed by imaging with a chemiluminescence imaging system (Vilber Fusion FX7, VILBER).

### IFA and confocal microscopy

The monolayer cells were washed with pre-chilled PBS, followed by fixation with 4% PFA for 10 min. The cells were then treated with 0.1% Triton X-100 in PBS at RT for 5 min and washed three times with PBS. Next, the cells were blocked with 5% bovine serum albumin (BSA) in PBS at RT for 1 h. After blocking, the cells were incubated with the appropriate primary antibodies at 4°C overnight and then treated with suitable fluorescent secondary antibodies. Cell nuclei were stained with DAPI for 5 min. The resulting fluorescent images were captured using a confocal laser scanning microscope (LSM700, Carl Zeiss AG). The images presented are representative slices taken from three independent experiments. Colocalization analyses were conducted utilizing the JaCoP plugin within ImageJ software, according to established protocols (67). The intensity distribution correlation between channels is described by Pearson’s correlation coefficient (>0.5). The Manders’ overlap coefficient (>0.6) reflected the extent of true colocalization. Meanwhile, the Manders’ colocalization coefficient represents the proportion of the colocalized fluorescence intensity of A protein with B protein to the total fluorescence intensity of A protein.

### FCM

The PRRSV-infected MARC-145 cells were washed with PBS, followed by digestion with 0.25% trypsin-EDTA at 37°C for 3 min. The digestion was immediately halted by adding the DMEM containing 10% FBS. The cells were then gently resuspended and centrifuged at 400 × *g* for 5 min, followed by two washes with PBS. Cell pellets were fixed in 4% PFA at RT for 10 min, permeabilized with 0.1% Triton X-100 for 5 min, and blocked with 5% BSA for 1 h. After blocking, the cells were incubated with mouse anti-PRRSV N MAb at RT for 2 h and then treated with Alexa Fluor 647-goat anti-mouse IgG fluorescent pAbs in the dark at RT for 1 h. The cells were centrifuged at 400 × *g* for 5 min and resuspended in 300 μL PBS. The percentage of PRRSV-infected cells was analyzed using a flow cytometer (CytoFLEX, Beckman Coulter).

### Attachment and internalization assay

The MARC-145 cells transfected with siEAP20 or siNC were infected with PRRSV (MOI = 10) and incubated at 4°C for 1 h to allow viral attachment. The unbound viral particles were removed through washing with pre-chilled PBS. For the attachment assay, the collected cells underwent RT-qPCR to quantify cell-bound viral RNA, and the viral titers were determined using the TCID₅₀ assay. Alternatively, after fixation, permeabilization, and blocking, the cells were stained with mouse anti-PRRSV GP5 MAb, and the cell-bound viruses were visualized using confocal microscopy. For the internalization assay, the cells pre-bound with PRRSV were shifted to 37°C for an additional 1 h to permit viral entry. The residual surface-bound particles were removed by thoroughly washing with PBS containing proteinase K. The intracellular RNA and infectious titers were then evaluated using RT-qPCR and TCID₅₀ assay, respectively. Alternatively, the cells were incubated with a mouse anti-PRRSV N MAb, and the internalized viruses were observed via confocal microscopy.

### TEM assay

The MARC-145 cells cultured in 60 mm dishes were transfected with siNC, siEAP20, or siEAP20 together with a plasmid harboring synonymous mutations in the *EAP20* silencing sites. At 36 hpt, the cells were infected with PRRSV at an MOI of 10. At 10 hpi, the cells underwent three washes with pre-chilled PBS and were gently scraped into 1.5 mL centrifuge tubes. Samples were then centrifuged at 400 × *g* for 2 min at 4°C, and the resulting cell pellets were fixed in 2.5% glutaraldehyde at RT for 30 min. Further processing, including gradient ethanol dehydration, resin infiltration, embedding, polymerization, and ultrathin sectioning, was conducted by Servicebio Company. Ultrathin sections were examined using an 80 kV TEM platform (Ht7800/Ht7700, HITACHI), and representative images were captured for analysis.

### Co-IP

HEK-293T cells were seeded in six-well plates and transfected at 60%–80% confluence. The cells were co-transfected with 1.25 μg of the 3× Flag-EAP20 expression plasmid and 1.25 μg of individual HA-tagged PRRSV protein expression plasmids (HA-Nsp1α, HA-Nsp1β, HA-Nsp2, HA-Nsp4, HA-Nsp5, HA-Nsp7, HA-Nsp9-12, and HA-N). At 36 hpt, the cells were washed once with pre-chilled PBS and lysed on ice for at least 30 min using WB/IP lysis buffer supplemented with 1 mM protease inhibitor cocktail and 1 mM PMSF. Lysates were clarified by centrifugation at 12,000 × *g* for 15 min. The supernatants were incubated with 20 μL anti-Flag or anti-HA magnetic beads at 4°C overnight under gentle rotation. The beads were recovered using a magnetic rack and washed once with WB/IP lysis buffer, followed by five washes with TBS. The bead-bound immunocomplexes were resuspended in 2× SDS loading buffer and heat-denatured at 95°C for 10 min. Finally, the samples were subjected to SDS-PAGE and subsequent IB with mouse anti-HA or anti-Flag MAb.

### IP

MARC-145 cells were seeded in six-well plates and harvested at the indicated time points post-infection. After one wash with pre-chilled PBS, the cells were lysed on ice for at least 30 min in 500 μL of WB/IP lysis buffer containing the protease inhibitor cocktail and PMSF. Lysates were centrifuged at 12,000 × *g* at 4°C for 15 min, and 100 μL of the supernatants was reserved as the input samples. The remaining 400 μL were incubated with rabbit anti-EAP20 pAbs at RT for 1 h, followed by incubation with 25 μL BeyoMag protein G plus magnetic beads for 1 h. The immune complexes were captured magnetically, washed once with WB/IP lysis buffer, and five times with PBS. After washing, 50 μL of 2× SDS loading buffer was added to the complexes and boiled for 10 min. The samples were then subjected to IB assay to detect the potential interactions between endogenous EAP20 and endosomal markers (clathrin, Rab5, Rab7, Rab11).

### Enrichment of ER/GA proteins

MARC-145 cells were seeded in 100 mm dishes and infected with PRRSV (MOI = 10). At 10 hpi, the cells were washed once with pre-chilled PBS, harvested using a scraper, and pelleted by centrifugation at 600 × *g* for 5 min. The obtained cell precipitates were stored at −80°C until use. The ER and GA fractions were isolated using a Minute ER/GA Enrichment Kit per the manufacturer’s instructions (68, 69).

### Statistical analysis

All experiments were performed in triplicate and independently repeated at least three times. Data are presented as means ± standard error of mean (SEM). Statistical analyses were evaluated using GraphPad Prism software (Version 8.0) by unpaired two-tailed Student’s *t*-tests for two-group comparisons or one-way analysis of variance (ANOVA) for multiple-group comparisons. Statistical significance values are denoted by asterisks in the figures (*, *P* < 0.05; **, *P* < 0.01; ***, *P* < 0.001; ****, *P* < 0.0001; ns [not significant], *P* > 0.05).

## Data Availability

The data supporting the findings of this study are available from the corresponding author upon reasonable request.
